# Estrogen Induces Mammary Ductal Dysplasia via the Upregulation of Myc Expression in a DNA-Repair-Deficient Condition

**DOI:** 10.1016/j.isci.2020.100821

**Published:** 2020-01-09

**Authors:** Junji Itou, Rei Takahashi, Hiroyuki Sasanuma, Masataka Tsuda, Suguru Morimoto, Yoshiaki Matsumoto, Tomoko Ishii, Fumiaki Sato, Shunichi Takeda, Masakazu Toi

**Affiliations:** 1 Laboratory of Molecular Life Science, Institute for Biomedical Research and Innovation, Foundation for Biomedical Research and Innovation at Kobe (FBRI), 2-2 Minatojima-Minamimachi, Chuo-ku, Kobe 650-0047, Japan; 2 Department of Breast Surgery, Graduate School of Medicine, Kyoto University, 54 Shogoin-Kawahara-cho, Sakyo-ku, Kyoto 606-8507, Japan; 3 Graduate School of Pharmaceutical Sciences and Faculty of Pharmaceutical Sciences, Doshisha Women's College of Liberal Arts, 97-1 Kodo, Kyotanabe 610-0395, Japan; 4 Department of Radiation Genetics, Graduate School of Medicine, Kyoto University, Yoshida-Konoe-cho, Kyoto 606-8501, Japan; 5 Program of Mathematical and Life Science, Graduate School of Integrated Sciences for Life, Hiroshima University, 1-3-1 Kagamiyama, Higashi-Hiroshima 739-8526, Japan; 6 Department of Breast Surgery, Kansai Electric Power Hospital & Kansai Electric Power Medical Research Institute, 2-1-7 Fukushima, Fukushima-ku, Osaka 553-0003, Japan

**Keywords:** Molecular Biology, Cell Biology, Cancer

## Abstract

Mammary ductal dysplasia is a phenotype observed in precancerous lesions and early-stage breast cancer. However, the mechanism of dysplasia formation remains elusive. Here we show, by establishing a novel dysplasia model system, that estrogen, a female hormone, has the potential to cause mammary ductal dysplasia. We injected estradiol (E2), the most active form of estrogen, daily into scid mice with a defect in non-homologous end joining repair and observed dysplasia formation with cell proliferation at day 30. The protooncogene *Myc* is a downstream target of estrogen signaling, and we found that its expression is augmented in mammary epithelial cells in this dysplasia model. Treatment with a Myc inhibitor reduced E2-induced dysplasia formation. Moreover, we found that isoflavones inhibited E2-induced dysplasia formation. Our dysplasia model system provides insights into the mechanistic understanding of breast tumorigenesis and the development of breast cancer prevention.

## Highlights

• Excess amount of estrogen administration in scid mice induces mammary ductal dysplasia
• E2-induced Myc expression is one of the causes of dysplasia formation
• Progesterone and isoflavones have a potential to prevent E2-induced dysplasia

## Graphical Abstract

**Figure:**
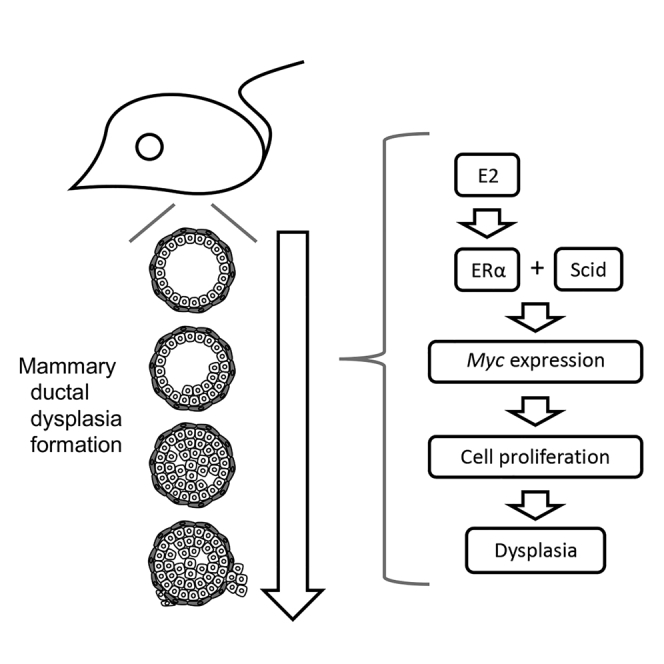

## Introduction

During breast tumorigenesis, mammary ductal dysplasia is observed in precancerous lesions and early-stage breast cancers. Mammary ductal dysplasia exhibits a loss of the biphasic mammary epithelial and myoepithelial pattern, an abnormal nucleus, epithelial cell expansion, a disruption in the myoepithelial cell layer, and/or mammary epithelial cell invasion to fibrous stroma, whereas a normal mammary duct maintains cell polarization, the biphasic pattern, and the smooth luminal surface of the mammary epithelial cell layer. Given that dysplasia can progress to malignant neoplasms (Arpino et al., 2005, Cichon et al., 2010, Sgroi, 2010), elucidating the mechanism of dysplasia formation will contribute to the prevention of breast tumorigenesis.

Previous studies have established various breast cancer mouse models by genetic engineering. For example, the mammary gland-specific expression of *c-neu* (*Her2*/*ErbB2*), *polyoma middle T* antigen, and *Wnt-1* causes breast cancer (Guy et al., 1992, Li et al., 2000, Muller et al., 1988). Mice with a mutation in *Tp53*, a tumor suppressor gene, also developed breast cancer (Kuperwasser et al., 2000). Although these models have provided knowledge about breast cancer, particularly at an advanced stage, they have not been primarily used for studies on mammary ductal dysplasia observed in precancerous lesions and early-stage breast cancers. Previous dysplasia studies in mice used radiation in *ataxia telangiectasia mutated* heterozygous mice (Weil et al., 2001) and the overexpression of constitutively activated Smoothened receptor (Moraes et al., 2007) and of the oncogene *nuclear receptor-binding SET domain protein 3* (Turner-Ivey et al., 2017). Because studies using genetically engineered mice have been designed to examine phenotypes specifically caused by the functions of their target genes and these studies could not provide knowledge about the relationships between the factors involved, these models are not suitable to elucidate the molecular mechanism by which mammary ductal dysplasia is naturally formed. To understand such a mechanism of dysplasia formation, a mouse model that forms dysplasia by physiological factor(s) and facilitates the mechanistic understanding of dysplasia formation is required.

Estrogen, a female hormone, promotes the development of the normal mammary duct and the proliferation of breast cancer (reviews Deroo and Korach, 2006, Heldring et al., 2007, Liang and Shang, 2013, Manavathi et al., 2013). There are a number of studies on the function of estrogen in breast cancer (reviews Burns and Korach, 2012, Deroo and Korach, 2006, Liang and Shang, 2013, Manavathi et al., 2013, Siersbæk et al., 2018). Although estrogen is thought to be involved in breast tumorigenesis in epidemiologic studies (Dall and Britt, 2017, Kelsey et al., 1993) and a combination of radiation and E2 treatment transformed normal mammary cells *in vitro* (Calaf and Hei, 2000), there is no experimental evidence showing that estrogen receptor α (ERα)-mediated estrogen signaling induces mammary dysplasia from normal mammary epithelial cells *in vivo*.

Estrogen regulates gene expression via the activation of ERα. In mammary glands, ERα is expressed mainly in mammary epithelial cells. Although there are various functional models of ERα action, in the classical mechanism of ERα, estrogen-bound ERα forms a dimer, localizes in the nucleus, and binds to its DNA-binding site to regulate gene expression (reviews Arnal et al., 2017, Burns and Korach, 2012, Heldring et al., 2007, Maggi, 2011, Yaşar et al., 2017). Thus far, various ERα-regulated genes have been identified (Liang and Shang, 2013, Manavathi et al., 2013, Siersbæk et al., 2018); these include *growth regulating estrogen receptor-binding 1* (*GREB1*), *trefoil factor 1* (*TFF1*, *pS2*), and *myelocytomatosis* (*MYC*) (Barkhem et al., 2002, Lin et al., 2004, Wang et al., 2011). *Myc* expression is associated with poor survival in breast cancer (Deming et al., 2000, Green et al., 2016). Myc (c-Myc) is also involved in breast cancer proliferation (Hart et al., 2014, Liao and Dickson, 2000, Liao et al., 2000). During ERα-mediated gene regulation, a DNA double-strand break is made at the promoter of a target gene to promote its expression (Ju et al., 2006, Williamson and Lees-Miller, 2011). Whether ERα-mediated gene regulation with a DNA double-strand break is involved in dysplasia formation remains unclear. In this study, we successfully established a novel dysplasia model system and investigated the mechanism of dysplasia formation.

## Results

### Enhancement of ERα-Mediated Estrogen Signaling Causes Mammary Ductal Dysplasia

To investigate E2-induced DNA damage *in vitro*, we treated an ERα-positive breast cancer cell line, MCF-7, with or without estradiol (E2), the most active form of estrogen, for 2 h and counted the number of the signals of phosphorylated-histone H2AX (gamma-H2AX, gH2AX), a marker of DNA double-strand breaks (Figure S1A). The number of gH2AX signals was increased by E2 treatment and reduced by cotreatment with fulvestrant, an estrogen receptor inhibitor (Figure S1A), indicating that E2 treatment causes DNA double-strand breaks via its receptor. An inhibitor of DNA-dependent protein kinase (DNA-PK), NU-7441, inhibits non-homologous end joining repair, one of the repair mechanisms of DNA double-strand breaks (Leahy et al., 2004). NU-7441 cotreatment did not change the number of gH2AX signals after 2-h E2 treatment (Figure S1A), indicating that the loss of DNA-PK function does not change E2-generated DNA damage under our conditions.

**Figure 1:**
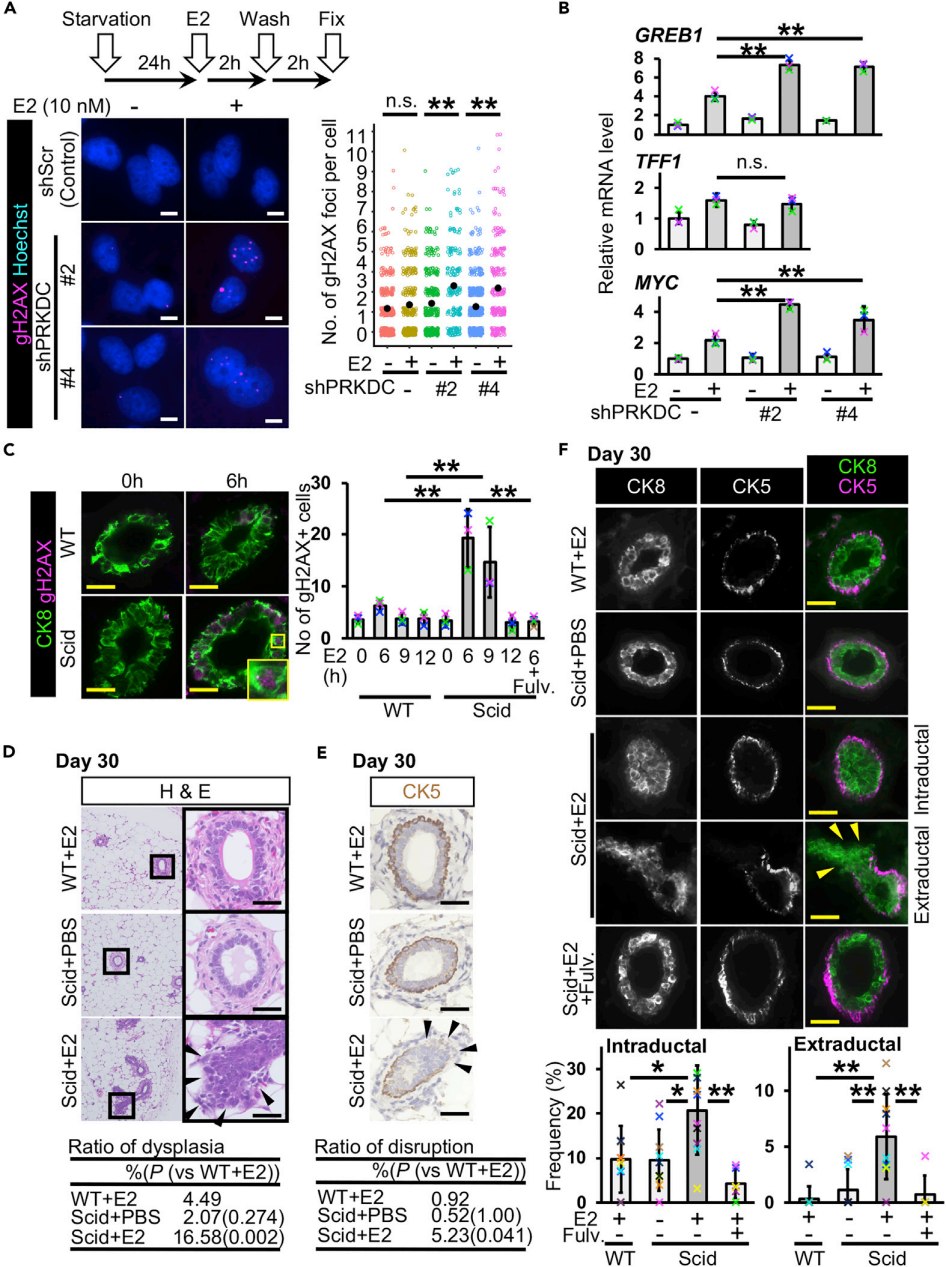
Estrogen Administration Induces Mammary Ductal Dysplasia in scid Mice (A) DNA double-strand breaks were detected in MCF-7 cells. *PRKDC* was knocked down. Gamma-H2AX was immunostained. Numbers of gH2AX foci per cell were graphed (jitter plot). Black dots indicate mean values. Data were obtained from 2 or 3 independent experiments (total 200–520 cells in each group, U Mann-Whitney test). (B) Messenger RNA levels of *GREB1*, *TFF1,* and *MYC* were quantified (*n* = 3 experiments, one-way ANOVA followed by Tukey's test). Cells were treated with or without E2 for 6 h. (C) Gamma-H2AX-positive mammary epithelial cells were detected and quantified at 6, 9, and 12 h after E2 administration (*n* = 3 mice in wild-type [WT] + E2 0 h, 6 h, 9 h, 12 h and scid + E2 0 h, 6 h, 9 h, 12 h; *n* = 4 mice in scid + E2 + fulvestrant 6 h, one-way ANOVA followed by Tukey's test). (D) Typical images of H&E staining are shown. Daily injection of E2 was performed for 30 days. The table shows ratios of dysplasia (*n* = 6 mice [one image from each mouse, total six images], WT + E2 4.49%, scid + PBS 2.07% [p = 0.274, versus WT + E2], and scid + E2 16.58% [p = 0.002, versus WT + E2], U Mann-Whitney test). (E) Typical immunostaining images of CK5 are shown. The table shows ratios of disruption (*n* = 6 mice [one image from each mouse, total six images], WT + E2 0.92%, scid + PBS 0.52% [p = 1.00, versus WT + E2], and scid + E2 5.23% [p = 0.041, versus WT + E2], U Mann-Whitney test). (F) Fluorescent images of CK8 and CK5 staining are shown. Mammary ducts with intraductal and extraductal expansion were quantified (*n* = 10 mice in WT + E2, scid + PBS and scid + E2 groups, *n* = 6 mice in scid + E2+Fulv. group, one-way ANOVA followed by Tukey's test and U Mann-Whitney test). Fulv., fulvestrant. Scale bars, 10 μm in (A) and 30 μm in (C–F). n.s., not significant, *p < 0.05, **p < 0.01. Error bars represent standard deviation. Arrowheads indicate mammary epithelial cells in extraductal region (D–F). In the graphs, crosses with different colors indicated the values of different samples (B) and animals (C and F).

To determine whether estrogen-induced DNA double-strand breaks are involved in the regulation of the downstream genes of estrogen signaling, we reduced the capacity of non-homologous end joining by knocking down *PRKDC*, which encodes the catalytic subunit of DNA-PK (Davis et al., 2014). Cells were washed with medium in the absence of E2 after 2-h E2 treatment to analyze repair capacity. No increase in the number of gH2AX signals was observed in control cells (Figure 1A), indicating that cells were allowed to repair E2-induced DNA double-strand breaks during 2 h after washing out E2 under our conditions. However, larger number of the gH2AX signals were observed after washing in *PRKDC*-knockdown cells than in control cells (Figures 1A and S1B), suggesting that the loss of DNA-PK function delays the repair of E2-induced DNA double-strand breaks. Long-lived DNA breaks (unrepaired at least 12 h after DNA damage induction) are believed to be involved in transcriptional regulation, at least in specific cases (Puc et al., 2017). To investigate the expression of ERα downstream genes under our conditions, mRNA levels were quantified in cells with or without 6-h E2 treatment (Figure 1B). *PRKDC* knockdown increased *GREB1* expression in untreated cells. In E2-treated groups, the expression of *GREB1* was enhanced following *PRKDC* knockdown. The expression of *TFF1* was not altered by *PRKDC* knockdown. Interestingly, after E2 treatment, we observed 2-fold higher *MYC* expression in *PRKDC*-knockdown cells than in control cells. We treated cells with NU-7441 and L189, an inhibitor for ligases I, III, and IV, to reduce the capacity of non-homologous end joining (Figure S1D). Ligases I, III, and IV are involved in DNA repair including non-homologous end joining. In cells treated with these inhibitors, higher *Myc* expression was observed after E2 stimulation than in DMSO-treated control cells (Figure S1E). These results suggest that a reduction in the repair capacity of DNA double-strand breaks enhances *MYC* expression in response to E2 stimulation.

We injected E2 (6 μg/mouse) intraperitoneally into scid mice with a genetic defect in DNA-PK catalytic subunit function (Bosma et al., 1983, Kirchgessner et al., 1995). The serum concentration of E2 was transiently increased and reduced to the background level after 6 h in both wild-type and scid mice (Figure S1C). The results of gH2AX staining showed an increased number of gH2AX-positive mammary epithelial cells (Figure 1C). As expected, scid mice showed a larger number of gH2AX-positive cells than wild-type mice at 6 h (5-fold larger) and 9 h (3-fold larger) after E2 administration. No increase in the number of gH2AX-positive mammary epithelial cells was observed at 9 h in wild-type and at 12 h in scid mice, suggesting that E2-induced DNA double-strand breaks were repaired at these time points, and E2 administration transiently induced DNA double-strand breaks under our conditions. Increase in the number of gH2AX-positive mammary epithelial cells was not observed in mice administered E2 and fulvestrant.

To investigate whether long-term E2 administration could cause an abnormality in the mammary gland, we performed consecutive daily injections of E2 for 30 days. No significant change in body weights was observed after 30 days of injection (Figure S2A). Hematoxylin and eosin (H&E) staining showed normal mammary ducts in both wild-type and scid mice at day 7 (Figure S2B). At day 30, whereas E2-injected wild-type (WT + E2) and phosphate-buffered saline (PBS)-injected scid (scid + PBS) mice exhibited normal mammary ducts (Figure 1D), dysplasia formation (i.e., increased cell number, loss of the biphasic mammary epithelial and myoepithelial pattern, and epithelial cell expansion to the outside of a duct) was observed in E2-injected scid mice (scid + E2) (Figures 1D and S2C). To examine whether mammary ducts with dysplasia had disrupted basement membrane, we coimmunostained for Laminin, the component of basement membrane, and CK8, the mammary epithelial cell marker. Some mammary ducts with dysplasia showed disrupted basement membrane and mammary epithelial cell invasion (Figure S2D). Because human breast cancer shows disruption in the myoepithelial layer (Cichon et al., 2010), we immunostained cells for the myoepithelial cell markers cytokeratin 5 (CK5) (Figure 1E) and p63 (Figure S2E). We observed a disruption in the myoepithelial layers in mammary ducts with dysplasia. To determine whether mammary epithelial cells of the ducts with dysplasia invaded the outside of the myoepithelial layer, coimmunostaining of CK5 and CK8 was performed (Figure 1F). The results revealed mammary ducts with increased mammary epithelial cell numbers and the loss of the biphasic pattern (Figure 1F intraductal) and mammary ducts with epithelial cells in the extraductal region with a disruption in the CK5 cell layer (Figure 1F extraductal) in scid + E2. As terminal end buds are epithelial cell-rich regions that can be identified as a duct with no fibrous stroma region (Sternlicht, 2006) and as it is difficult to distinguish dysplasia from normal terminal end buds, these were excluded in the analyses. We analyzed the frequency of dysplasia formation and observed significant increases in both intraductal and extraductal expansion (Figure 1F graph, Table S1). A duct with both intraductal and extraductal expansion was counted as a duct with extraductal expansion. These results suggest that repetitive E2 administration induces mammary ductal dysplasia in scid mice. Coadministration with the estrogen receptor inhibitor (scid + E2 + Fulv.) showed no increase in the frequency of dysplasia, suggesting that the suppression of estrogen receptor function may prevent dysplasia formation in a DNA repair-deficient condition. ERα expression was observed in mammary epithelial cells in both normal and dysplastic ducts (Figure S2F). ERα positivity was not only difference between PBS and E2 administration but also between normal and dysplastic ducts in scid mice.

We coadministered NU-7441, a DNA-PK inhibitor, and E2 to two wild-type strains, C.B17/Icr and C57BL/6J. C.B17/Icr is the parental strain of scid mice. C57BL/6J is a commonly used strain. At day 30, we observed an increase in dysplasia formation in NU-7441 + E2 mice compared with NU-7441 + PBS mice in both strains (Figure S3 and Table S1). This result indicates that E2 administration can induce mammary ductal dysplasia not only in scid mice but also in wild-type strains with induced DNA repair defect.

### Progesterone Inhibits E2-Induced Dysplasia Formation

**Figure 2:**
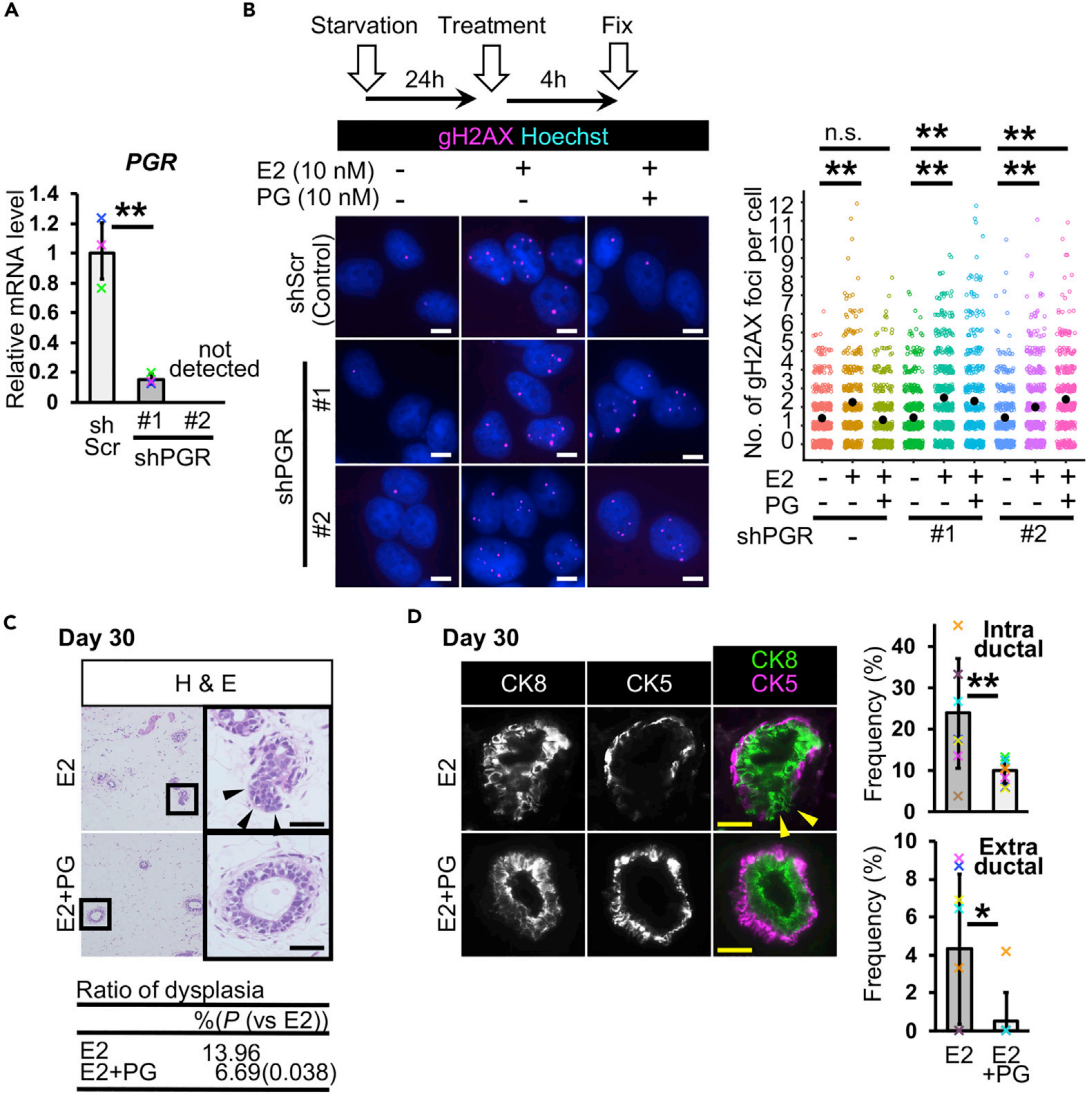
Progesterone Inhibits Estrogen-Induced Mammary Ductal Dysplasia (A) Progesterone receptor (*PGR*) gene was knocked down (*n* = 3 experiments, Student's t test to shScr control). (B) DNA double-strand breaks were detected by gH2AX immunostaining. Numbers of gH2AX foci per cell were analyzed (jitter plot) (*n* = 3 independent experiments, total 380–520 cells in each group, U Mann-Whitney test). (C) Typical images of H&E staining are shown. The table shows ratios of dysplasia (*n* = 6 mice [one image from each mouse, total six images], E2 13.96% and E2 + PG 6.69% [p = 0.038, versus E2], U Mann-Whitney test). (D) Fluorescent images of CK8 and CK5 staining are shown. Mammary ducts with intraductal and extraductal expansion were quantified (*n* = 8 mice, U Mann-Whitney test). Scale bars, 10 μm in (B) and 30 μm in (C and D). n.s., not significant, *p < 0.05, **p < 0.01. Error bars represent standard deviation. Arrowheads indicate mammary epithelial cells in extraductal region (C and D). In the graphs, crosses with different colors indicated the values of different samples (A) and animals (D).

Progesterone (PG), a female hormone, alters ERα chromatin binding events in malignant breast cancer through progesterone receptor (PGR), which changes gene expression patterns, and the administration of PG reduced E2-dependent tumor growth in mouse xenograft experiments with MCF-7 cells (Mohammed et al., 2015). To determine whether PG-activated PGR inhibits E2-induced DNA double-strand breaks in MCF-7 cells, we performed a combination treatment of E2 and PG for 4 h with or without *PGR* knockdown (Figures 2A and 2B). The number of gH2AX signals was increased by E2 treatment but not by the combination of E2 and PG (Figure 2B). In *PGR*-knockdown cells, the combination treatment of E2 and PG resulted in increased number of gH2AX signals, similar to E2 treatment, suggesting that PGR prevents E2-induced DNA double-strand breaks.

The function of PG in dysplasia formation *in vivo* remains elusive. To this end, we coinjected E2 and PG into our dysplasia model system. In scid mice at day 30, the administration of E2 alone caused dysplasia formation, and the coadministration of E2 and PG prevented dysplasia formation (Figure 2C). Double immunostaining for CK5 and CK8 showed that PG administration prevented extraductal expansion (Figure 2D). The quantification of intraductal and extraductal expansion showed that PG reduced the frequencies of these abnormalities (Figure 2D). These results suggest that PG has a protective effect against E2-induced DNA damage and dysplasia formation in a DNA-repair-deficient condition.

### Estrogen Promotes Mammary Epithelial Cell Proliferation

**Figure 3:**
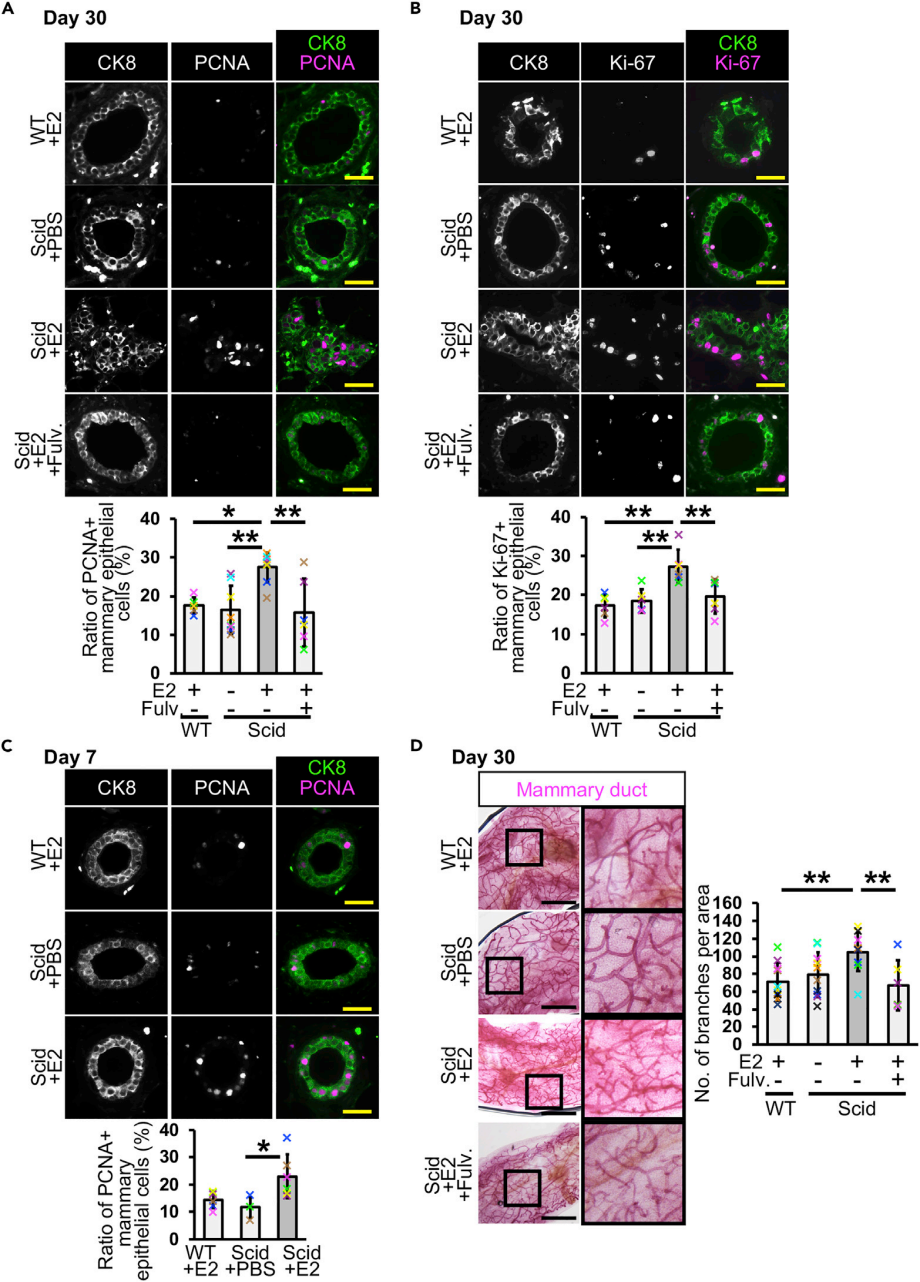
Estrogen Administration Promotes Mammary Epithelial Cell Proliferation in scid Mice (A) PCNA-positive mammary epithelial cells were detected at day 30. Ratios of the positive cells were analyzed (*n* = 6 mice in WT + E2 and scid + E2 + Fulv. groups, and *n* = 8 mice in scid + PBS and scid + E2 groups, one-way ANOVA followed by Tukey's test). (B) Ki-67-positive mammary epithelial cells were detected. Ratios of the positive cells were analyzed (*n* = 6 mice, one-way ANOVA followed by Tukey's test). (C) PCNA-positive cells were analyzed in day 7 mice (*n* = 6 mice in WT + E2 and scid + E2 groups, and *n* = 3 in scid + PBS group, one-way ANOVA followed by Tukey's test). (D) Carmine alum staining was performed in mammary ducts of day 30 mice. Numbers of branches in 9 mm^2^ area close to lymph node were counted (*n* = 10 mice in WT + E2, scid + PBS, and scid + E2 groups, *n* = 6 mice in scid + E2 + Fulv. group, one-way ANOVA followed by Tukey's test). Fulv., fulvestrant. Scale bars, 30 μm in (A–C) and 2 mm in (D). *p < 0.05, **p < 0.01. Error bars represent standard deviation. In the graphs, crosses with different colors indicated the values of different animals.

As the number of CK8-positive cells was likely increased in scid + E2 mice (Figure 1F), we investigated cell proliferation by immunostaining for proliferating cell nuclear antigen (PCNA), an S-phase marker, and Ki-67, a proliferation marker, at day 30 (Figures 3A and 3B). The results of immunostaining showed that the ratio of proliferating mammary epithelial cells was increased 1.5-fold in scid + E2 mice, and this increase was inhibited by the ERα inhibitor (scid + E2+Fulv.). An increased number of proliferating cells was also observed at day 7 (Figures S4 and 3C). To investigate organ-level changes, we visualized mammary ducts with carmine alum staining at day 30 (Figure 3D). The mammary glands of scid + E2 mice were more densely distributed than those of the control mice and exhibited more small branches. The number of branches was increased in scid + E2 mice compared with control mice (Figure 3D graph). These results suggest that E2 administration induces cell proliferation in the mammary glands of scid mice.

### E2-Induced Myc Expression Causes Mammary Ductal Dysplasia

**Figure 4:**
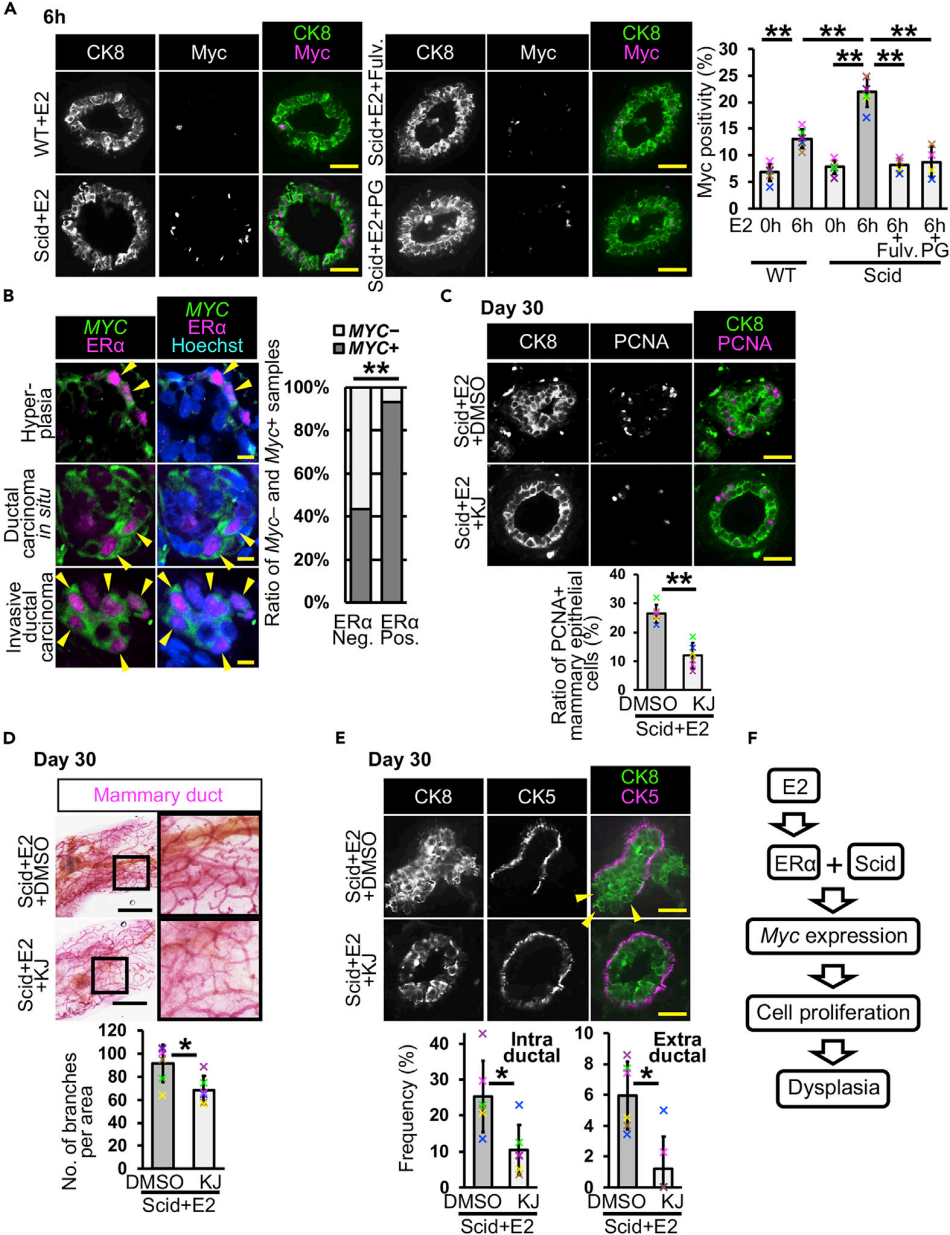
Estrogen-Induced Myc Expression Promotes Mammary Epithelial Cell Proliferation and Dysplasia Formation (A) Typical images of Myc immunostaining are shown. Ratios of Myc-positive mammary epithelial cells are analyzed (*n* = 6 mice in WT + E2 and scid + E2, *n* = 4 mice in scid + E2 + Fulv. and scid + E2 + PG, one-way ANOVA followed by Tukey's test). (B) Typical images of the combination of *MYC in situ* hybridization and ERα immunostaining are shown. Arrowheads indicate *MYC*-expressing ERα-positive cells. *MYC* positivity was analyzed in the samples from malignant tumors (*n* = 39 ERα-negative and 29 ERα-positive samples, Fisher's exact test). (C) PCNA was detected in mammary glands of mice treated with or without an Myc inhibitor, KJ-Pyr-9. Ratio of PCNA-positive cells were quantified (*n* = 6 mice, Student's t test). (D–F) Typical images of carmine alum staining are shown. Numbers of branches in 9 mm^2^ area close to lymph node were counted (*n* = 6 mice, Student's t test). (E) Fluorescent images of CK8 and CK5 staining are shown. Mammary ducts with intraductal and extraductal expansion were quantified (*n* = 6 mice, U Mann-Whitney test). Arrowheads indicate mammary epithelial cells in extraductal region. (F) E2-induced *Myc* expression causes mammary ductal dysplasia in scid mice. Fulv.: fulvestrant, KJ, KJ-Pyr-9. Scale bars, 30 μm in (A, C, and E), 10 μm in (B), and 2 mm in (D). *p < 0.05, **p < 0.01. Error bars represent standard deviation. In the graphs, crosses with different colors indicated the values of different animals.

We hypothesized that one of the causes of dysplasia is the E2-induced expression of ERα downstream genes. Our *in vitro* experiments showed that E2 administration has the potential to increase the expression of a protooncogene, *MYC* (Figure 1B). In a previous study, mice with enhanced Myc expression exhibited mammary cell proliferation and an increase in branching (Tseng et al., 2014), similar to our observation in scid + E2 mice (Figure 3). We therefore focused on Myc. The results of *Myc* mRNA *in situ* hybridization showed that although *Myc* mRNA expression was increased at 2 h after E2 injection in the mammary ducts of both wild-type and scid mice, scid mice had stronger signals than did wild-type mice (Figure S5A). In scid mice, *Myc* mRNA expression was still clearly observed at 6 and 9 h and faintly at 12 and 24 h, whereas the signal was not observed at 6 h onward in wild-type mice (Figure S5A). In the results of gH2AX staining (Figure 1C), the number gH2AX-positive mammary epithelial cells were transiently increased at 6 and 9 h in scid mice. These results suggest that E2-induced double-strand breaks may promote *Myc* expression, but not elongate *Myc* expression for longer periods. A significant increase in the number of Myc-positive mammary epithelial cells was observed in scid mice at 6 h and day 30 (Figures 4A and S5B). This increase was not observed following the coadministration of E2 and fulvestrant or PG (Figure 4A). These results indicate that E2 administration increases Myc expression *in vivo*.

To determine whether estrogen signaling promotes *MYC* expression in human breast cancer, we analyzed the expression of *Myc* mRNA and ERα protein in a tissue microarray with human breast tissues. *Myc* mRNA was observed in the cytoplasm, and ERα was localized in the nucleus (Figure 4B). We observed *Myc* and ERα double-positive cells in the samples of hyperplasia, ductal carcinoma *in situ,* and invasive ductal carcinoma. Because of intratumoral heterogeneity, there were also *Myc*-positive ERα-negative, *Myc*-negative ERα-positive, and *Myc* and ERα double-negative cells. We analyzed the ratio of *Myc*-positive tissues in ERα-negative and ERα-positive malignant breast cancers, and *Myc* positivity was higher in ERα-positive tissues than in ERα-negative tissues (Figure 4B graph, negative 43.58% versus positive 93.10%). In *Myc*-positive ERα-positive tissues, 26 of 27 samples had *Myc* and ERα double-positive cells (96.29%). These observations suggest that estrogen signaling may be involved in the modulation of *Myc* expression in human breast cancer.

We coadministered E2 and the Myc inhibitor KJ-Pyr-9 (Hart et al., 2014), in scid mice, to investigate the involvement of Myc in dysplasia formation. We observed around a 2-fold reduction in the ratio of PCNA-positive mammary epithelial cells by Myc inhibition compared with the DMSO control at day 30 (Figure 4C). At the organ level, the number of branching points was also reduced (Figure 4D). These results indicate that Myc inhibition leads to reduced E2-induced cell proliferation in the mammary gland in scid mice.

Ductal dysplasia was investigated with H&E staining and immunostaining for CK5 and CK8 at day 30. Most mammary ducts (96.04%) were normal in the Myc inhibitor-administered group (Figure S5C). Moreover, intraductal and extraductal expansion was significantly reduced following administration of the Myc inhibitor (Figure 4E). The Myc inhibitor did not alter ERα positivity (Figure S5D). These results indicate that E2-induced Myc expression is one of the causes of mammary ductal dysplasia (Figure 4F).

### Isoflavones Prevent E2-Induced Dysplasia Formation

**Figure 5:**
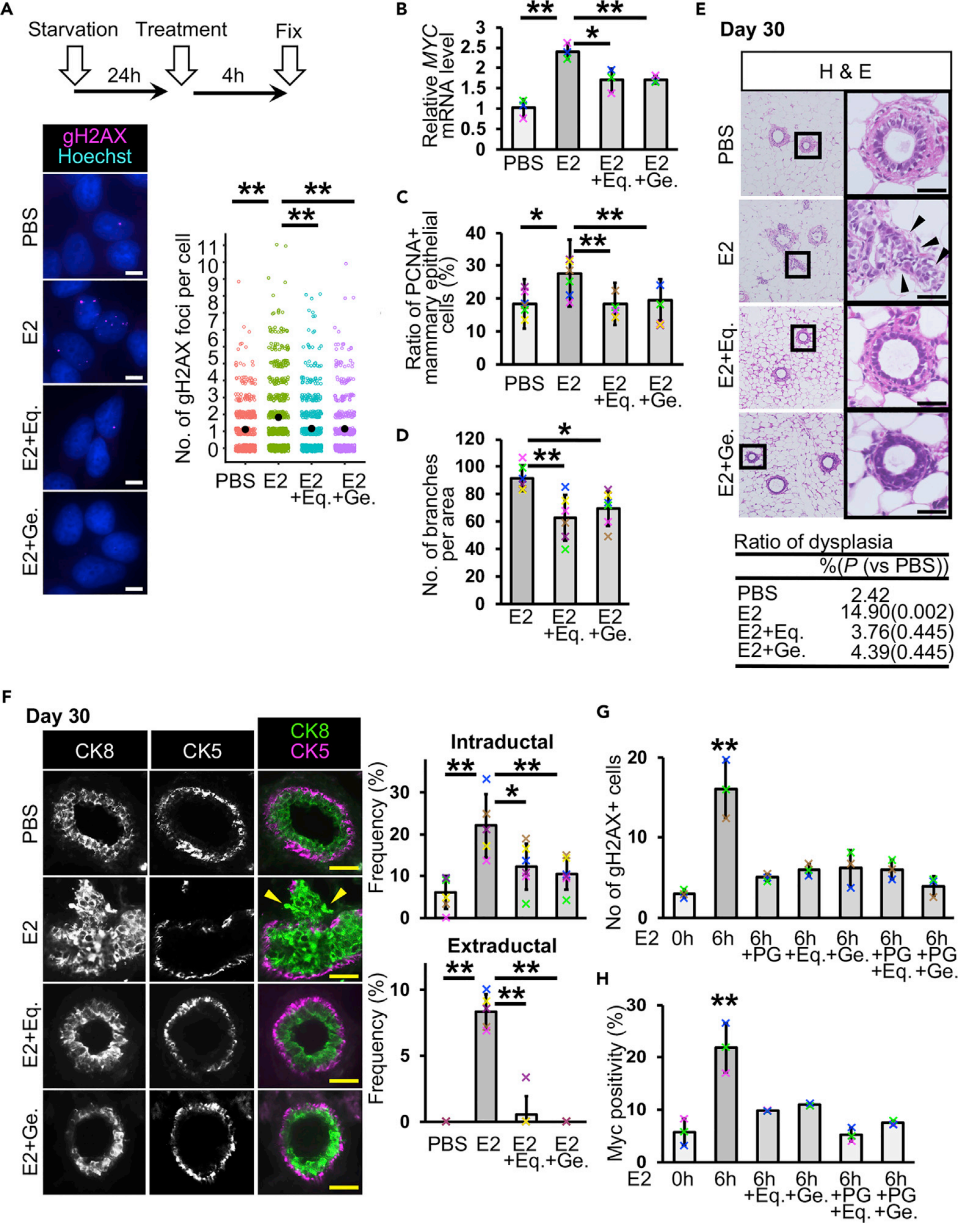
Isoflavones Inhibit Dysplasia Formation by Preventing the Function of Estrogen in scid Mice (A) DNA double-strand breaks were detected by gH2AX immunostaining. Numbers of gH2AX foci per cell were analyzed (jitter plot) (*n* = 3 independent experiments, total 550–670 cells in each group, U Mann-Whitney test). (B) *MYC* mRNA levels were quantified in MCF-7 cells (*n* = 3 experiments, one-way ANOVA followed by Tukey's test). (C) Ratios of PCNA-positive cells were analyzed (*n* = 6 mice, one-way ANOVA followed by Tukey's test). (D) Numbers of branches were analyzed in carmine alum-stained mammary glands (*n* = 6 mice, one-way ANOVA followed by Tukey's test). (E) Typical images of H&E staining are shown. The table shows ratios of dysplasia (*n* = 6 mice [one image from each mouse, total six images), PBS 2.42%, E2 14.90% [p = 0.002, versus PBS], E2 + Eq. 3.76% [p = 0.445, versus PBS], and E2 + Ge. 4.39% [p = 0.445, versus PBS], U Mann-Whitney test). (F) Fluorescent images of CK8 and CK5 staining are shown. Mammary ducts with intraductal and extraductal expansion were quantified (*n* = 6 mice in scid + PBS, scid + E2 + Eq., and scid + E2 + Ge. groups, *n* = 5 mice in scid + E2, one-way ANOVA followed by Tukey's test and U Mann-Whitney test). (G) Ratios of gH2AX-positive mammary epithelial cells were analyzed (*n* = 3 mice, one-way ANOVA followed by Tukey's test). (H) Ratios of Myc-positive mammary epithelial cells were analyzed (*n* = 3 mice, one-way ANOVA followed by Tukey's test). Eq, (S)-equol, Ge., genistein. Scale bars, 10 μm in (A) and 30 μm in (E and F). *p < 0.05, **p < 0.01. Error bars represent standard deviation. Arrowheads indicate mammary epithelial cells in extraductal region (E and F). In the graphs, crosses with different colors indicated the values of different samples (B) and animals (C, D, and F–H).

Our dysplasia model system can be utilized to study breast cancer prevention. Isoflavones are flavonoids and are rich in soybean. Epidemiological studies have suggested that isoflavones have a protective effect against breast cancer (Fritz et al., 2013). Genistein, an isoflavone, inhibited breast cancer cell growth (Shao et al., 1998). On the other hand, a study using breast cancer cells showed that genistein promoted tumor growth (Hsieh et al., 1998). E2 and isoflavones bound to estrogen receptors competitively, and isoflavone binding reduced estrogen receptor-mediated gene expression *in vitro* (Morito et al., 2001). Therefore, the effect of isoflavones in breast cancer is still controversial. To address this issue, we investigated the effect of isoflavones in our dysplasia model system. We used two isoflavones, (S)-equol and genistein. When E2-induced DNA double-strand breaks were analyzed, both isoflavones reduced E2-induced DNA damage (Figure 5A). *MYC* mRNA quantification showed that isoflavones reduced *MYC* expression 1.4-fold under E2-treated conditions compared with treatment with E2 alone, although its expression level was higher than in the PBS control (Figure 5B). These results suggest that isoflavones have the potential to reduce E2 function.

The coadministration of E2 and isoflavones reduced the proliferation of mammary epithelial cells in scid mice at day 30 (Figures 5C and S6A). The number of branches was reduced by isoflavones (Figures 5D and S6B). These results suggest that isoflavones are effective at preventing E2-induced cell proliferation. H&E staining showed that most of the mammary ducts of mice coadministered E2 and isoflavones had normal ductal structures and retained the biphasic mammary epithelial and myoepithelial pattern at day 30 (Figure 5E). The results of CK5 and CK8 immunostaining showed that the ratio of intraductal and extraductal expansion was reduced by isoflavone administration (Figure 5F). These results indicate that isoflavones have the potential to prevent E2-induced dysplasia formation in scid mice.

To investigate whether PG and isoflavones work in the same pathway to prevent dysplasia formation, we performed combination treatments of E2, PG, and isoflavones. In the results of gH2AX staining at 6 h, administration of PG and isoflavones in combination with E2 did not show increase in the number of gH2AX-positive mammary epithelial cells (Figures 5G and S6C). Myc positivity was reduced in the mice administered E2 + isoflavones, although the positivity was higher than control at 6 h (Figures 5H and S6D). This observation is consistent with our *in vitro* assays (Figure 5B). E2 + PG administration showed a comparable Myc positivity with control (Figure 4A). Combination treatment of E2 + PG + isoflavones also showed a similar Myc positivity to the control. These results suggest that although both PG and isoflavones can reduce E2-induced DNA double-strand breaks, the inhibitory mechanism of Myc expression was not the same.

### Progesterone, Myc Inhibitor, and Isoflavones Have a Potential to Prevent Mammary Ductal Dysplasia Formation in Wild-Type Mice

**Figure 6:**
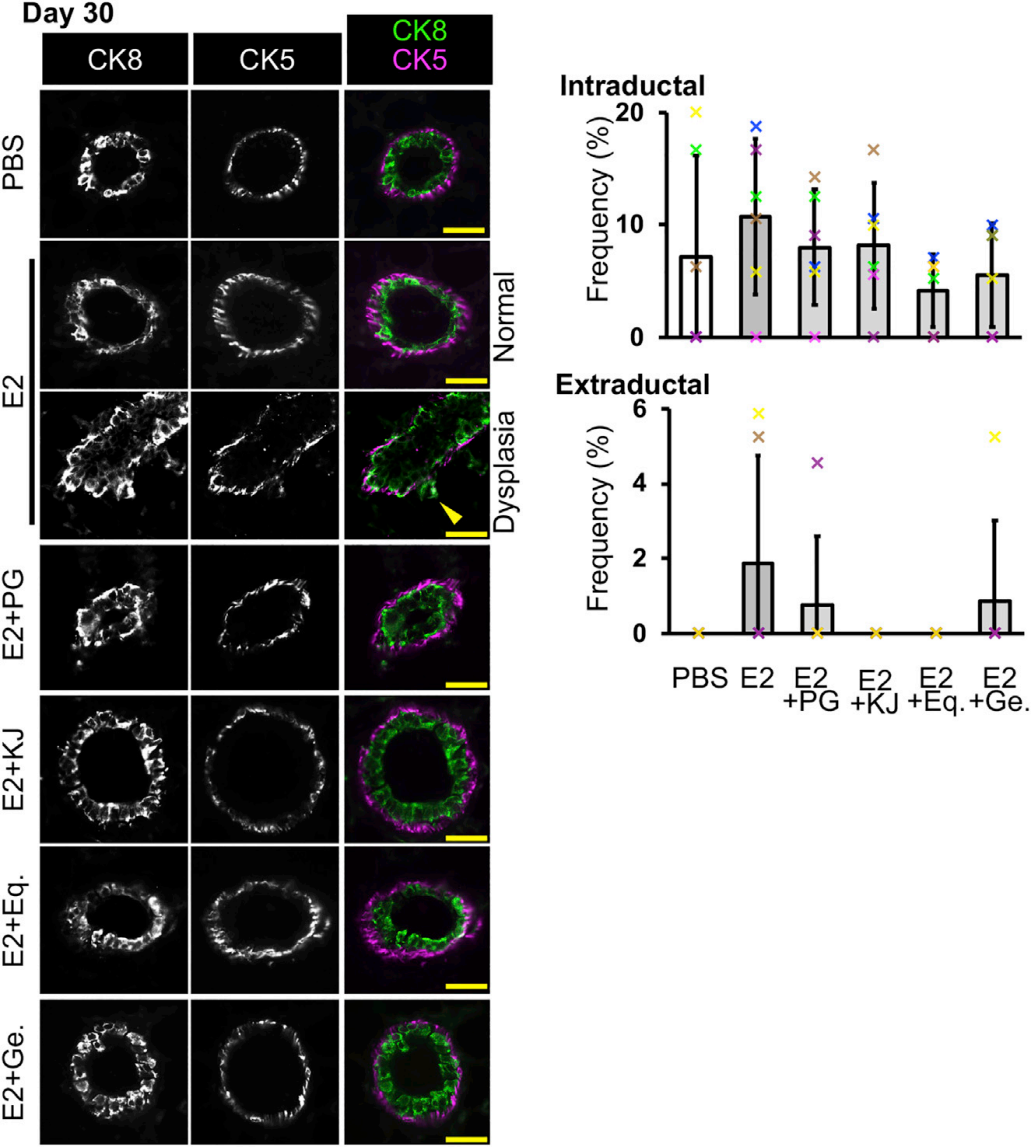
Progesterone, Myc Inhibitor, and Isoflavones Have a Potential to Reduce Mammary Dysplasia Formation C57BL/6J mice were administered E2 in combination with PG, KJ, Eq. or Ge. for 30 days. Fluorescent images of CK8 and CK5 staining are shown. Mammary ducts with intraductal and extraductal expansion were quantified (*n* = 6 mice, one-way ANOVA followed by Tukey's test and U Mann-Whitney test). Scale bars, 30 μm. Error bars represent standard deviation. In the graphs, crosses with different colors indicated the values of different animals. Eq., (S)-equol; Ge., genistein; KJ, KJ-Pyr-9.

To investigate the effects of PG, KJ-Pyr-9, (S)-equol, and genistein in wild-type mice without DNA repair deficiency, we coadministered E2 and these materials to C57BL/6J mice for 30 days. In the results of CK8 and CK5 double staining (Figure 6), mice administered E2 alone showed slight increase in the frequencies of intraductal and extraductal expansion, although the changes were not statistically significant between mice administered PBS and E2. This slight increase was prevented by coadministration with PG, KJ-Pyr-9, (S)-equol, and genistein. These results suggest that PG, Myc inhibitor, and isoflavones may have a potential to prevent E2-induced mammary ductal dysplasia.

## Discussion

We demonstrated E2-induced mammary ductal dysplasia in mice, providing direct evidence that estrogen causes mammary ductal dysplasia. This study showed that E2 administration in scid mice enhances *Myc* expression, which promotes dysplasia formation. In humans, long-term estrogen exposure has the potential to cause breast cancer (Dall and Britt, 2017, Kelsey et al., 1993). On the other hand, DNA repair capacity is not maintained throughout life (Kalfalah et al., 2015, Li et al., 2016). In the breast, human mammary epithelial cells from aged donors were more sensitive to mammography-induced DNA damage than cells from young donors (Hernández et al., 2013). Mammary epithelial cells from older women showed a delay in DNA double-strand break repair, compared with those from younger women (Anglada et al., 2019). Mutations in genes related to the DNA damage response/repair lead to the risk of developing breast cancer (Miki et al., 1994, Stankovic et al., 1998). Our dysplasia-inducing model system (i.e., 30-day E2 injection in mice with reduced DNA repair capacity) may mimic some of the situations of human breast tissue that are susceptible to mammary ductal dysplasia.

Although our dysplasia model uses an excess amount of E2 and mice with reduced DNA repair capacity, it will be useful to investigate the mechanism of dysplasia formation and to develop a method for dysplasia prevention. By using this system, we revealed that Myc is one of the causes of dysplasia formation. However, one question that remains is why does E2 promote Myc expression and cell proliferation in almost all parts of a mammary gland but dysplasia occurs in only a small portion. To address this question, the identification of other factors is required, which may be facilitated with our dysplasia model system. Although Myc expression alone is not sufficient to develop malignant breast tumors (Stewart et al., 1984, Tseng et al., 2014), given that Myc overexpression induces a replicative stress and stress-associated diseases, including cancer (Murga et al., 2011), E2-induced MYC expression may be an early event in breast tumorigenesis. In addition, we showed dysplasia formation in wild-type strains by coadministration of the DNA-PK inhibitor and E2. These results indicate that our dysplasia model system can be utilized in various strains and genetically engineered mouse models, such as *Tp53*-knockout mice.

We observed that the number of DNA double-strand breaks and *Myc* expression were increased in scid mice at 6 and 9 h after E2 administration. DNA double-strand breaks were repaired at 12 h. On the other hand, in scid mice, E2 administration did not immediately promote mammary epithelial cell proliferation, and the proliferation was observed at day 7. These results suggest that E2-induced DNA double-strand breaks and *Myc* expression are not directly involved in the initiation of cell proliferation, or in addition to *Myc* expression, mammary epithelial cell proliferation requires unidentified factor(s), which works at day 7 in our model system.

It is considered that breast cancer metastasizes in a very early stage in some cases (Fisher, 1980, Fisher et al., 1981), which causes distant metastasis and metastatic recurrence. Given that microinvasion is an early event in metastasis, inhibition of microinvasion in a very early stage may reduce breast cancer mortality. E2-induced dysplasia observed in this study showed microinvasion. Administration of Myc inhibitor or isoflavones inhibited it. These results suggest that our dysplasia model system can be utilized to study very-early-stage breast cancer for metastasis prevention.

For the breast cancer prevention study, we showed the first experimental evidence that isoflavones inhibited E2-induced dysplasia formation in a DNA repair-deficient condition. Although our dysplasia model system does not mimic all kinds of breast cancer types, our findings suggest that isoflavones can be utilized to prevent breast cancer. In the future, this dysplasia induction model system may contribute to the understanding of breast cancer tumorigenesis and to the development of breast cancer prevention.

### Limitation of the Study

Our dysplasia model system is a model for estrogen-induced dysplasia, but not for dysplasia caused by other factors, implying that our findings contribute to the understanding of the tumorigenesis of some breast cancer types, although early stages of most breast cancer types exhibit dysplasia with similar morphological changes.

## Methods

All methods can be found in the accompanying Transparent Methods supplemental file.

## Supplemental Information

Document S1. Transparent Methods, Figures S1–S6, and Table S1

Table S2. p values, Related to Figures 1, 2, 3, 4, 5, 6, and S1–S5
