# Supplementary material for: Estrogen Induces Mammary Ductal Dysplasia via the Upregulation of Myc Expression in a DNA-Repair-Deficient Condition
Source: iScience. 2020 Jan 9;23(2):100821. doi: 10.1016/j.isci.2020.100821 (PMC6976935; doi:10.1016/j.isci.2020.100821)
Supplement: Document S1. Transparent Methods, Figures S1–S6, and Table S1 [file mmc1.pdf]

iScience, Volume *23*

Supplemental Information

Estrogen Induces Mammary Ductal Dysplasia via the Upregulation of Myc Expression in a DNA-Repair-Deficient Condition

Junji Itou, Rei Takahashi, Hiroyuki Sasanuma, Masataka Tsuda, Suguru Morimoto, Yoshiaki Matsumoto, Tomoko Ishii, Fumiaki Sato, Shunichi Takeda, and Masakazu Toi

Estrogen induces mammary ductal dysplasia via the upregulation of Myc expression in a DNA-repair deficient condition.

Junji Itou, Rei Takahashi, Hiroyuki Sasanuma, Masataka Tsuda, Suguru Morimoto, Yoshiaki Matsumoto, Tomoko Ishii, Fumiaki Sato, Shunichi Takeda, Masakazu Toi

Transparent Methods

Supplemental figures

Figure S1 Estrogen administration induces DNA double-strand breaks. Related to Figure 1

Figure S2 Long-term estrogen administration causes dysplasia. Related to Figure 1

Figure S3 Dysplasia is induced by the combination of E2 administration and DNA-PK pharmacological inhibition. Related to Figure 1

Figure S4 Mammary epithelial cell proliferation is observed at day 7 in the dysplasia model system. Related to Figure 3

Figure S5 Estrogen administration induces Myc expression *in vivo* in mammary epithelial cells. Related to Figure 4

Figure S6 Isoflavones inhibit estrogen-induced cell proliferation in the mammary gland. Related to Figure 5

Supplemental table

Table S1 Frequency of dysplasia formation, related to Figure 1, S3

Transparent Methods

Cell culture

MCF-7 cells were obtained from the American Type Culture Collection (Manassas, VA, USA). Short tandem repeat analysis was performed for cell authentication in July 2017 and the result showed no contamination and no alteration. Mycoplasma contamination was checked every 3 months by staining with Hoechst 33342 (Dojindo, 346-07951, Kamimashiki, Japan, 1/500 dilution) and no contamination was observed. Cells were maintained in Roswell Park Memorial Institute 1640 (RPMI-1640) medium containing 10% heat-inactivated fetal bovine serum (FBS), 100 units/mL Penicillin, 100 μg/mL Streptomycin and 1nM β-estradiol (E2) (Sigma, E2758, St. Louis, MO, USA) at 37°C with 5% CO_2. To obtain G1 phase cells For gH2AX staining experiments, cells were starved with phenol-red-free FBS-free RPMI-1640 medium containing 100 units/mL Penicillin and 100 μg/mL Streptomycin for 24 h. E2, progesterone (PG) (Sigma, P8783), (S)-equol (Cayman Chemical, 10010173, Ann Arbor, MI, USA) and genistein (Nagara Science, NH010302, Gifu, Japan) were solved in ethanol, diluted with PBS and added to medium (final concentration: 10 nM). Fulvestrant (Sigma, I4409) was solved with ethanol, diluted with PBS and added to medium (final concentration: 100 nM). NU-7441 (AdooQ Bioscience, A11098, Irvine, CA, USA) was solved in DMSO, diluted with PBS and added to medium (final concentration: 0.5 μM). For mRNA quantification of ERα downstream genes, cells were cultured with phenol-red-free RPMI-1640 medium containing 10% charcoal-stripped FBS, 100 units/mL Penicillin and 100 μg/mL Streptomycin for 48 h, subsequently treated with or without 10 nM E2 for 6 h.

Loss of function study

For short hair-pin RNA (shRNA) expression, a lentiviral vector pLKO.1 (Addgene, 8453, Cambridge, MA, USA) was used. Double-strand DNA oligo with shRNA sequence was cloned into the region between *Age*I and *Eco*RI sites of the vector. The target sequences were 5'-CCAGTGAAAGTCTGAATCATT-3' (shPRKDC #2), 5'-CCTGAAGTCTTTACAACATAT-3' (shPRKDC #4), 5'-GCTGCTGGAAGACGAAAGTTA-3' (shPGR #1) and 5'-CAATACAGCTTCGAGTCATTA-3' (shPGR #2). Control shRNA was 5'-CCTAAGGTTAAGTCGCCCTCG-3' (shScr).

Lentiviral vector was cotransfected with lentiviral envelope and packaging

plasmids, pMDLg/pRRE (Addgene, 12251), pMD2.G (Addgene, 12259) and pRSV-Rev (Addgene, 12253) at a ratio of 2.5:1.0:0.6:0.5 into Lenti-X 293T cells (Takara, 632180, Kusatsu, Japan). The transfection reagent, FuGENE 6 (Promega, E2691, Madison, WI, USA) was used. Lenti-X 293T cells were maintained with Dulbecco's modified Eagle medium containing 10% FBS, 100 units/mL Penicillin and 100 μg/mL Streptomycin. One day after transfection, medium was changed to the medium for MCF-7 cells and incubated for 24~30 h. Medium containing lentiviral particles was filtered (0.22 μm pore size), added to MCF-7 cell culture with 6 μg/mL polybrene and incubated for 48 h. For drug selection to obtain infectants, cells were treated with 1 μg/mL puromycin for 4 days.

Messenger RNA quantification

Cells were cultured in 6-well plate or 6 cm dish. Cells were rinsed with cold PBS and treated with 300 μL of Trizol reagent (Thermo Fisher Scientific, 15596018, Waltham, MA, USA). Sixty μL of chloroform was added, mixed and stand for 5 min. After centrifugation at 4°C, supernatant was collected and purified with PureLink RNA Micro Kit (Thermo Fisher Scientific, 12183016).

Five hundred ng of total RNA was used for complementary DNA (cDNA) synthesis. SuperScript III reverse transcriptase (Thermo Fisher Scientific, 18080044) was used. Synthesized cDNA was diluted with sterilized MilliQ water (1/10 dilution) for real-time polymerase chain reaction (PCR).

Real-time PCR was performed with a reagent, FastStart Universal SYBR Green Master (Sigma, 04 913 850 001, St. Louis, MO, USA). Signals were detected by StepOnePlus real-time PCR system (Thermo Fisher Scientific, 4376600) with StepOne software ver2.2.2.

Primer sequences were: *EF1A1* (internal control) forward: 5'-AAATGACCCACCAATGGAAGCAGC-3' reverse: 5'-TGAGCCGTGTGGCAATCCAATACA-3', *PRKDC* forward: 5'-CGCCGTGTGAATATAAAGATTGG-3' reverse: 5'-CGTGACTGTTTCAGTACGATTAG-3', *GREB1* forward: 5'-CTGCTGTACCTCTGTGACTCTT-3' reverse: 5'-GTCCTGACAGATGACACACAAC-3', *TFF1* forward: 5'-CCCTGGTCCTGGTGTCCAT-3' reverse: 5'-AGCAGCCCTTATTTGCACACT-3', *MYC* forward: 5'-CTCGGATTCTCTGCTCTCCT-3' reverse: 5'-TCTTCCTCATCTTCTTGTTCCTC-3', *PGR* forward: 5'-

CACAGCGTTTCTATCAACTTACAA-3' reverse: 5'-CCGGGACTGGATAAATGTATTC-3'.

Immunostaining in cell culture

Cells were plated onto an 8-well chamber slide (Matsunami glass, SCS-N08, Kishiwada, Japan) with 400 μL medium, and cultured for 2 days. For fixation, a half of the medium was removed, and 200 μL of 4% paraformaldehyde (PFA) in PBS was added (final 2% PFA). After 10~15 min fixation at room-temperature, cells were washed with PBS containing 0.05% Tween-20 (PBS-T). Permeabilization was performed with PBS containing 0.1% Triton-X 100 for 15 min at room-temperature. Cells were washed with PBS-T twice. Blocking was performed with 5% goat serum-containing PBS-T for 1 h at room-temperature. Cells were incubated with primary antibody in blocking solution for overnight (15~20 h) at 4°C. Primary antibody was anti-gH2AX antibody (S139) (Cell Signaling Technology, 2577S, Danvers, MA, USA, 1/200 dilution). Cells were washed with PBS-T 3 times and incubated with secondary antibody in blocking solution for 1~2 h at room-temperature. Secondary antibody was goat anti-rabbit IgG antibody conjugated to Alexa Fluor 546 (Life Technologies, A11010, 1/1000 dilution). Cells were washed with PBS twice and counterstained with Hoechst 33342 for 30 min at room-temperature. Cells were washed with PBS, dried and mounted with Fluoromount-G (SouthernBiotech, 0100-01, Birmingham, AL, USA).

Mouse experiments

Female C.B17/Icr wild-type (C.B17/Icr-scidJcl +/+) and scid (C.B17/Icr-scidJcl scid/scid) mice were purchased from CLEA Japan (Tokyo, Japan) (6~8-week-old). Six-week-old female C57BL/6J mice were purchased from Japan SLC (Hamamatsu, Japan). Mice were maintained under specific- pathogen-free condition. Intraperitoneal injection was performed with 30G needle in the morning. Injected reagents were E2 (6 μg/day), PG (6 μg/day), Fulvestrant (100 μg/day), NU-7441 (100 μg/day), KJ-Pyr-9 (Namiki Shoji, HY-19735, Tokyo, Japan, 0.2 mg/day), (S)-equol (6 μg/day) and genistein (6 μg/day). Mice were euthanized by cervical dislocation, and mammary glands were isolated. For 30-day samples, mammary glands were isolated at 24 h after final injection. For measurement of E2 serum concentration after administration, 5-week-old female mice were ovariectomized to eliminate endogenous E2. After 5 weeks, E2 was injected

intraperitoneally, and blood samples were collected from tail vein. E2 concentration was measured by using E2 ELISA(EIA) kit (Calbiotech, ES180S-100, El Cajon, CA, USA), SPECTRA max 340PC (Molecular devices, San Jose, CA, USA) and SoftMax Pro 5.4 (Molecular Devices). The animal experiments were approved by the Animal Research Committee of Kyoto University, number MedKyo17554 and MedKyo18321. All animals were maintained according to the Guide for the Care and Use of Laboratory Animals (National Institute of Health Publication).

Immunostaining in mammary gland

For cryo-section, isolated mammary gland was fixed with 4% PFA in PBS shortly (4 °C, 15 min, rocking). the sample was washed with PBS 3 times and incubated with 30% sucrose in PBS for 1~2 h at room-temperature. the sample was embedded in OCT compound (Sakura Finetek, 4583, Tokyo, Japan), and frozen with liquid nitrogen. Ten μm cryo-section was cut at -50°C, and dried. Dried section was fixed with 4% PFA for 3 min at room-temperature and rinsed with PBS. For paraffin-section, mammary gland was fixed with 10% formaldehyde neutral buffer solution for longer than 24 h at room-temperature, dehydrated and embedded in paraffin. Three μm paraffin-section was de-paraffinized, washed with PBS and rinsed with H_2O. For heat-induced epitope retrieval, specimen was put into boiling sodium citrate buffer (10 mM sodium citrate, 0.05 % Tween-20, pH 6.0), incubated for 40 min and cooled for 20 min.

Specimen was washed with PBS-T 3 times and blocked with blocking solution (5% goat serum in PBS-T) for 1 h at room-temperature. Specimen was incubated with primary antibody in blocking solution for overnight (15~20 h) at 4 °C. Primary antibodies were anti-gH2AX antibody (Cell Signaling Technology, 2577S, 1/200 dilution), anti-CK8 antibody (Developmental Studies Hybridoma Bank, TROMA-I, Iowa City, IA, USA 1/200 dilution), anti-CK5 antibody (Abcam, ab75869, 1/200 dilution), anti-Laminin antibody (Sigma, L9393, 1/500 dilution), anti-PCNA antibody clone PC10 conjugated to Alexa Fluor 647 (BioLegend, 307912, San Diego, CA, 1/20 dilution), anti-Ki-67 antibody D3B5 (Cell Signaling Technology, 12202S, 1/400 dilution), anti-c-Myc antibody (Abcam, ab32072, 1/200 dilution), anti-ERα antibody (Millipore, 06-935, 1/400 dilution) and anti-ERα antibody SP1 (NeoMarkers, RM-9101-S0, Fremont, CA, USA, 1/100 dilution). Specimen was washed 3 times with PBS-T and incubated with secondary antibody in blocking solution. Secondary antibodies were biotinylated goat anti-rabbit IgG (Vector,

BA-1000, Burlingame, CA, USA, 1/200 dilution), goat anti-rat IgG conjugated to Alexa Fluor 488 (Cell Signaling Technology, 4416S, 1/1000 dilution) and goat anti-rabbit IgG antibody conjugated to Alexa Fluor 546 (Life Technologies, A11010, 1/1000 dilution).

For DAB colorimetric detection, after secondary antibody reaction, specimen was blocked with 3% H_2O_2 for 10 min, washed with PBS 3 times and incubated with ABC kit (Vector, PK-6101). Specimen was washed with PBS and rinsed with H_2O. After DAB reaction, the specimen was washed with H_2O and counterstained with hematoxylin. The specimen was rinsed with H_2O and washed with H_2O for 10 min. Dehydrated specimen was mounted with Malinol (Muto Pure Chemicals, 2009-1, Tokyo, Japan).

For immunofluorescence, after secondary antibody reaction, specimen was washed with PBS twice and counterstained with Hoechst 33342 for 30 min at room-temperature. The specimen was washed with PBS, dried and mounted with Fluoromount-G. In each animal, 3 or 4 sections were analyzed.

Carmine Alum-staining

Isolated mammary gland was fixed with 4% PFA for 2 h at 4°C, washed with PBS twice and washed with H_2O. The sample was incubated with Carmine Alum staining solution (2 mg/mL carmine, 5 mg/mL aluminum potassium sulfate, a small amount of thymol) for overnight (20~24 h) at room-temperature. After staining, the sample was washed with 70% ethanol for 1 h at room-temperature, 95% ethanol for 1 h at room-temperature and 100% ethanol for 1 h at room-temperature. Subsequently the sample was cleared with xylene overnight (16~20 h) at room-temperature. Xylene was replaced to methyl salicylate for storage. One image was taken in each animal and numbers of branches in 9 mm^2 area close to lymph node were counted.

In situ *hybridization*

For probe synthesis, DIG RNA labeling kit (SP6/T7) (Roche, 11 175 025 910, Mannjeim, Germany) was used. The kit contains a vector, pSPT18, and RNA polymerases. A probe for mouse *Myc* mRNA was used (Itou et al., 2012). The coding sequence of human *MYC* gene was amplified from the cDNA sample of MCF-7 cells. *Myc* and *MYC* genes were cloned into the region between the *Hind*III and the *Eco*RI sites of the pSPT18 vector. Linearized *Myc* and *MYC* vectors were obtained by cutting with *Hind*III and *Pst*I, respectively. T7 RNA polymerase was used. Synthesized RNA probe was purified and

diluted with hybridization solution (50% formamide, 5x SSC, 5x Denharts, 250 μg/mL yeast tRNA, 500 μg/mL salmon sperm DNA).

For cryo-section, section 18 μm thick was dried overnight. A 4 μm thick paraffin-section of the tissue microarray of human breast tissues obtained with patient's informed consent (US Biomax, BRC1502, Derwood, MD, USA) was purchased and de-paraffinized. Investigations were performed according to the principles expressed in the Declaration of Helsinki.

Section was fixed with 4% PFA in PBS for 10 min at room-temperature and washed with PBS for 3 min 3 times. The section was treated with 2 μg/mL proteinase K in PBS for 10 min at room-temperature and fixed with 4% PFA in PBS for 5 min at room-temperature. The section was washed with PBS for 3 min 3 times. For acetylation, the section was put into a solution (295 mL H_2O, 4 mL triethanolamine and 0.525 mL HCl), then 0.75 mL acetic anhydrate was added, and mixed by dipping. After 10 min acetylation at room-temperature, the section was washed with PBS for 3 min 3 times. PBS was removed, and the section was incubated with hybridization solution for 1 h at room-temperature. Probe solution (30 ng probe in 100 μL hybridization solution) was prepared, heated at 80°C for 5 min, and iced. Hybridization was performed for overnight (18~20 h) at 68°C.

After hybridization, the section was washed with 5x SSC for 10 min at 65°C. Subsequently the section was washed with 0.2x SSC for 1 h at 65°C 3 times, washed with 0.2x SSC for 5 min at room-temperature and washed with TBS solution (0.1M Tris-HCl pH7.5, 0.15M NaCl) for 5 min at room-temperature. Blocking was performed with a blocking solution, 10% goat serum in TBS, for 1 h at room-temperature. After blocking, anti-DIG antibody conjugated to alkaline phosphatase (Roche, 11 093 274 910, 1/5000 dilution) or anti-DIG antibody conjugated to Fluorescein (Roche, 11 207 741 910, 1/200 dilution) was diluted in TBS solution containing 1% goat serum, and the section was incubated with the antibody solution at 4°C for overnight (18~22 h). For combination with immunostaining, a primary antibody was added to the antibody solution.

For alkaline phosphatase colorimetric reaction, section was washed with TBS solution for 5 min at room-temperature 3 times. The section was equilibrated with a solution (0.1M Tris-HCl pH9.5, 0.1M NaCl, 0.05M $MgCl_2$). Color development was performed with NBT/BCIP solution (Roche, 11 681 451 001) for 4~8 h at room-temperature. Reaction was stopped by washing with PBS. The section was fixed with 4%

PFA in PBS for 20 min at room-temperature. The section was washed with H_2O, dried and mounted with MX oil (Matsunami glass, FX00100). In mouse experiments, 3 or 4 sections were analyzed in each animal.

For combination with *in situ* hybridization and immunostaining, after primary antibody reaction, the section was washed with PBS-T for 5 min 3 times. Incubated with secondary antibodies in 5% goat serum-containing PBS-T. Secondary antibody for *in situ* hybridization was anti-fluorescein/Oregon Green antibody conjugated to Alexa Fluor 488 (Life technologies, A11096, 1/1000 dilution). The section was washed with PBS twice and counterstained with Hoechst 33342 for 30 min at room-temperature. The section was washed with PBS, dried and mounted with Fluoromount-G.

Microscopy

Images of H&E staining, immunostaining and *in situ* hybridization were collected at room temperature with an all-in-one microscope BZ-9000 (Keyence, Osaka, Japan) equipped with a 20x plan apochromatic objective lens (NA: 0.75), a x40 plan apochromatic lens (NA: 0.95) and a x100 plan apochromatic lens (NA: 1.40), and BZ-II Viewer software (Keyence). Hoechst 33342 signal was excited by 340-380 nm light and detected with 435-485 nm light. Alexa Fluor 488 signal was excited by 450-490 nm light and detected with 510-560 nm light. Alexa Fluor 546 signal was excited by 527.5-552.5 nm light and detected with 577.5-632.5 nm light. Alexa Fluor 647 signal was excited by 590-650 nm light and detected with 662.5-737.5 nm light.

Images of Carmine Alum-stained mammary glands were collected at room-temperature with a stereoscope, SMZ800 (Nikon, Tokyo, Japan) and Digital Sight DS-Fi1 (Nikon)

Statistical analyses

Numbers of gH2AX foci of cultured cells were counted manually and analyzed by U Mann-Whitney test. Delta Ct values of real-time PCR experiments were normalized by the mean values of the control groups and analyzed by student's *t*-test and one-way ANOVA followed by Tukey's test. Numbers of mammary epithelial cells having more than 5 gH2AX foci were counted manually and analyzed by one-way ANOVA followed by Tukey's test. Values of E2 serum concentration were normalized by the mean values of 0 h samples and analyze by student's *t*-test between wild-type and scid mice in each

time point. Numbers of mammary ducts with intraductal and extraductal expansion were analyzed by one-way ANOVA followed by Tukey's test and U Mann-Whitney test. Numbers of mammary epithelial cells having immunostaining signals of PCNA, Ki-67, Myc and ERα were counted manually, and analyzed by one-way ANOVA followed by Tukey's test and student's *t*-test. Numbers of branches were analyzed by one-way ANOVA followed by Tukey's test and student's *t*-test. *MYC* positivity of ERα negative and positive groups in the tissue microarray was analyzed by Fisher's exact test. *P* values are listed in Table S2. $P<0.05$ was considered to be statistically significant.

Supplemental reference

Itou, J., Kawakami, H., Quach, T., Osterwalder, M., Evans, S.M., Zeller, R., and Kawakami, Y. (2012). Islet1 regulates establishment of the posterior hindlimb field upstream of the Hand2-Shh morphoregulatory gene network in mouse embryos. Development (Cambridge, England) *139*, 1620-1629.

Key resource table

Reagent or resource	Source	Identifier
**Antibodies**		
gH2AX	Cell Signaling Technology	2577S
CK8	Developmental Studies Hybridoma Bank	TROMA-I
CK5	Abcam	ab75869
Laminin	Sigma	L9393
PCNA	BioLegend	307912
Ki-67	Cell Signaling Technology	12202S
c-Myc	Abcam	ab32072
ERα	Millipore	06-935
ERα	NeoMarkers	RM-9101-S0
**Biological Samples**		
Human breast tissue microarray	US Biomax	BRC1502
**Chemicals, Peptides, and Recombinant Proteines**		
Hoechst 33342	Dojindo	346-07951

β-estradiol	Sigma	E2758
Progesterone	Sigma	P8783
(S)-equol	Cayman Chemical	10010173
Genistein	Nagara Science	NH010302
Fulvestrant	Sigma	I4409
NU-7441	AdooQ Bioscience	A11098
L189	Cayman Chemical	18374
KJ-Pyr-9	Namiki Shoji	HY-19735
**Critical Commercial Assays**		
FuGENE 6	Promega	E2691
Trizol	Thermo Fisher Scientific	15596018
PureLink RNA Micro Kit	Thermo Fisher Scientific	12183016
SuperScript III	Thermo Fisher Scientific	18080044
FastStart Universal SYBR Green Master	Sigma	04 913 850 001
E2 ELISA(EIA) kit	Calbiotech	ES180S-100
DIG RNA labeling kit	Roche	11 175 025 910
**Experimental models: Cell lines**		
MCF-7	ATCC	HTB-22
Lenti-X 293T	Takara	632180
**Experimental models: Organisms/Strains**		
Mouse: C.B17/Icr-scidJcl +/+	CLEA Japan	N/A
Mouse: C.B17/Icr-scidJcl scid/scid	CLEA Japan	N/A
Mouse: C57BL/6J	Japan SLC	N/A
**Oligonucleotides**		
Primer: *EF1A1* forward AAATGACCCACCAATGGAAGCAGC	This paper	N/A
Primer: *EF1A1* reverse TGAGCCGTGTGGCAATCCAATACA	This paper	N/A
Primer: *PRKDC* forward CGCCGTGTGAATATAAAGATTGG	This paper	N/A
Primer: *PRKDC* reverse CGTGACTGTTTCAGTACGATTAG	This paper	N/A

Primer: *GREB1* forward CTGCTGTACCTCTGTGACTCTT	This paper	N/A
Primer: *GREB1* reverse GTCCTGACAGATGACACACAAC	This paper	N/A
Primer: *TFF1* forward CCCTGGTCCTGGTGTCCAT	This paper	N/A
Primer: *TFF1* reverse AGCAGCCCTTATTTGCACACT	This paper	N/A
Primer: *MYC* forward CTCGGATTCTCTGCTCTCCT	This paper	N/A
Primer: *MYC* reverse TCTTCCTCATCTTCTTGTTCCTC	This paper	N/A
Primer: *PGR* forward CACAGCGTTTCTATCAACTTACAA	This paper	N/A
Primer: *PGR* reverse CCGGGACTGGATAAATGTATTC	This paper	N/A
**Recombinant DNA**		
Plasmid: pLKO.1	Addgene	8453
Plasmid: pMDLg/pRRE	Addgene	12251
Plasmid: pMD2.G	Addgene	12259
Plasmid: pRSV-Rev	Addgene	12253
**Software and Algorithms**		
ImageJ	NIH	https://imagej.nih.gov/ij/
R	R Development Core Team	https://cran.r-project.org/
JMP	JMP Inc.	Ver 14.0.0

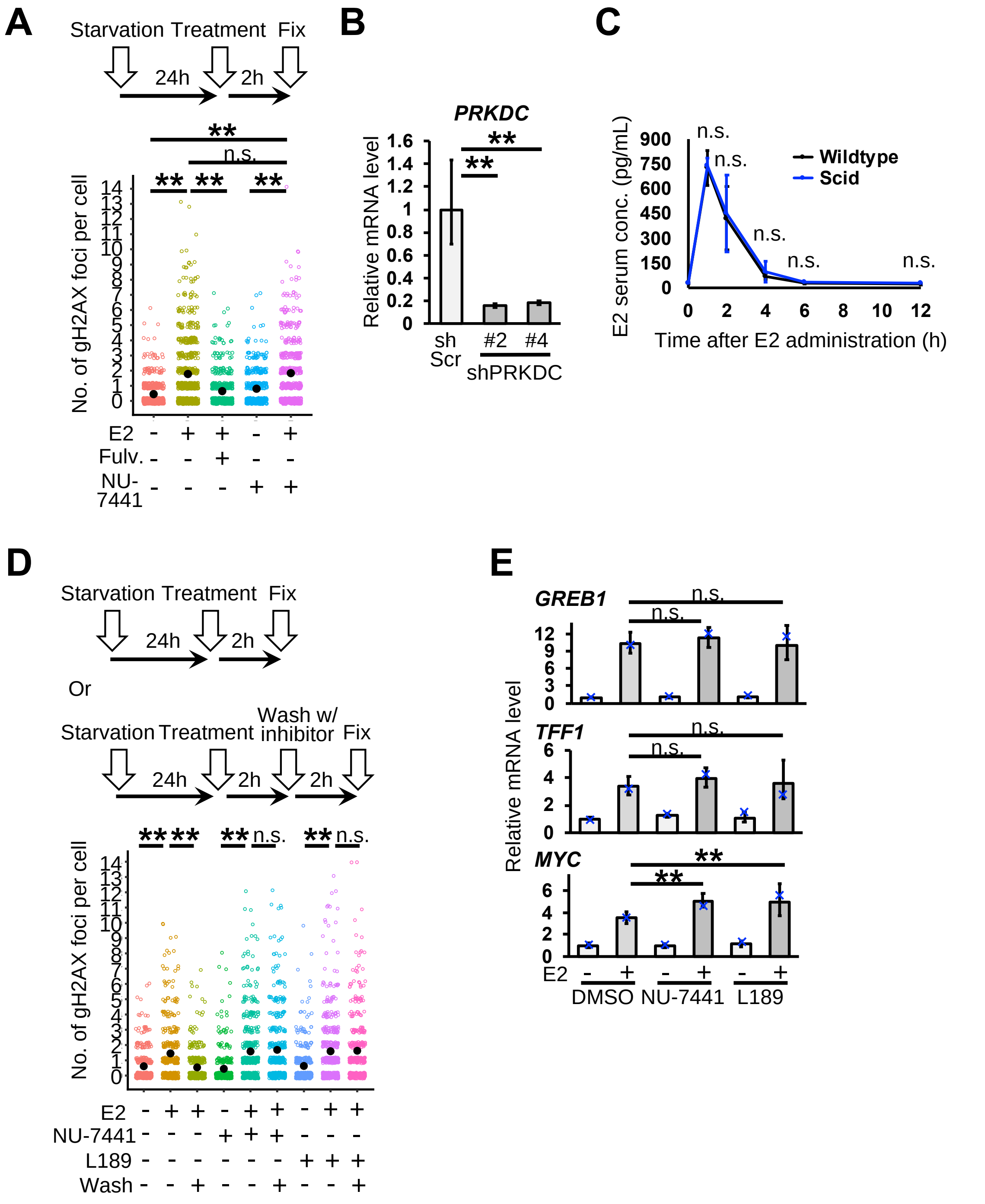

Figure S1 Estrogen administration induces DNA double-strand breaks. Related to Figure 1

A, DNA double-strand breaks were detected in MCF-7 cells. Numbers of gH2AX foci per cell were graphed (jitter plot). Black dots indicate mean values (total 459~760 cells in each group, U Mann-Whitney test). Fulv.: fulvestrant, an estrogen receptor inhibitor.

NU-7441, a DNA-PK inhibitor. B, *PRKDC* gene was knocked-down (*n*=3 experiments, student's *t*-test to shScr control). MCF-7 cells were used. C, E2 serum concentration was measured (*n*=3 mice, student's *t*-test in each time point). D. Numbers of gH2AX foci per cell were graphed (jitter plot). Black dots indicate mean values (total 374~515 cells in each group, U Mann-Whitney test). Messenger RNA levels of *GREB1*, *TFF1* and *MYC* were quantified (*n*=3 experiments, one-way ANOVA followed by Tukey's test). n.s.: not significant, **: $P<0.01$. Error bars represent standard deviation. In the graphs, crosses with different colors indicated the values of different samples.

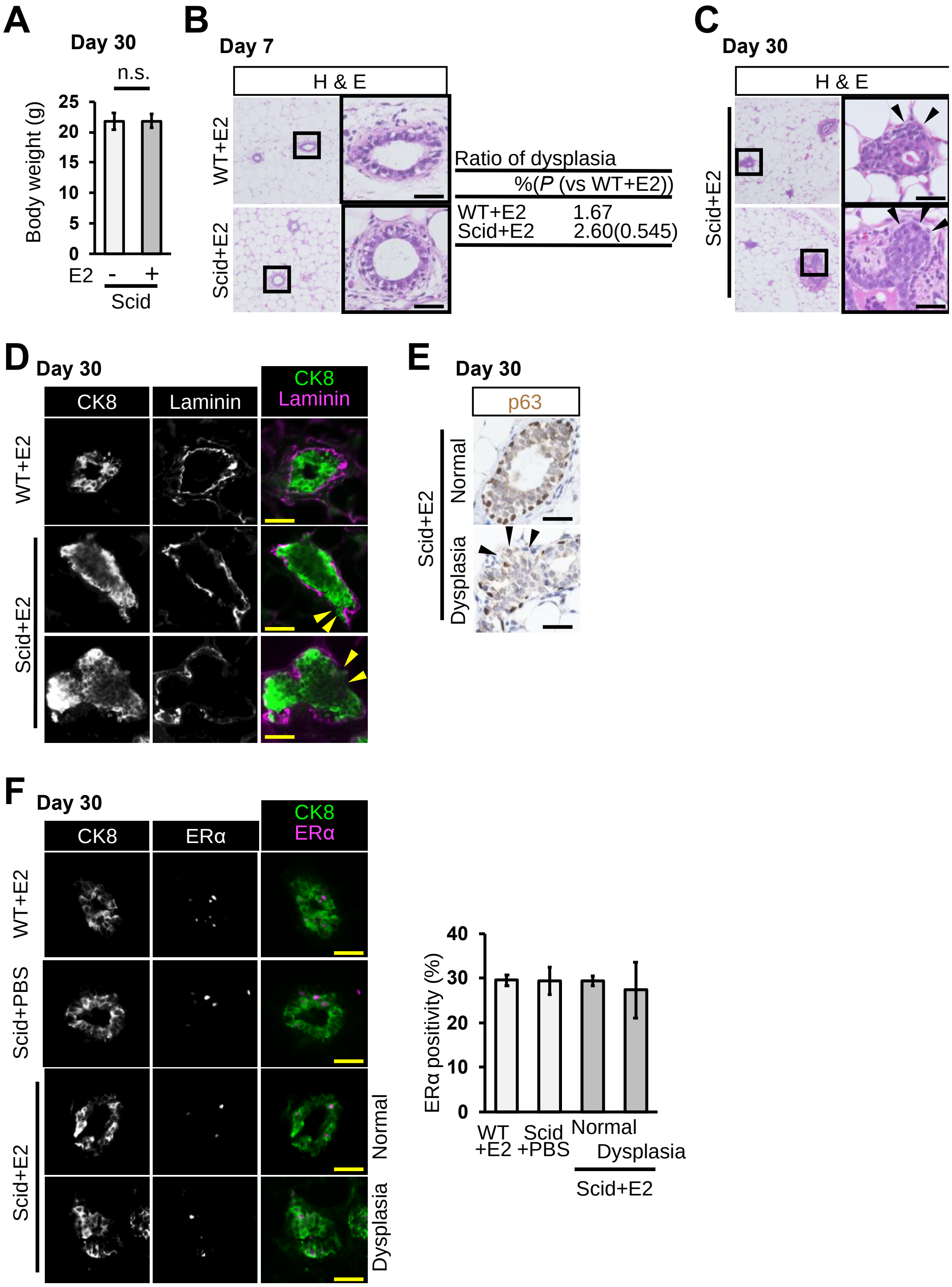

Figure S2 Long-term estrogen administration causes dysplasia. Related to Figure 1 A, Body weight was measured at day 30 (*n*=10 mice, student's *t*-test). B, Images of H&E staining of mammary glands are shown. Mammary glands were isolated at day 7. The table shows ratios of dysplasia (*n*=6 mice (one image from each mouse, total 6 images), WT+E2 1.67% and scid+E2 2.60% (*P*=0.545 (vs WT+E2)), U Mann-Whitney test). C,

Additional to Fig. 1D, H&E images of mammary glands of E2-administered scid mice are shown. Arrowheads indicate mammary epithelial cells in extraductal region. D, Basement membrane was stained with anti-Laminin antibody. Arrowheads indicate a region lost basement membrane. E, The myoepithelial marker, p63, was stained. Arrowheads indicate a region lost myoepithelial cells. F, ERα was immunostained. Ratios of ERα-positive mammary epithelial cells were analyzed (*n*=3 mice, one-way ANOVA followed by Tukey's test). Scale bars indicate 30 μm. n.s.: not significant. Error bars represent standard deviation. In the graphs, crosses with different colors indicated the values of different animals.

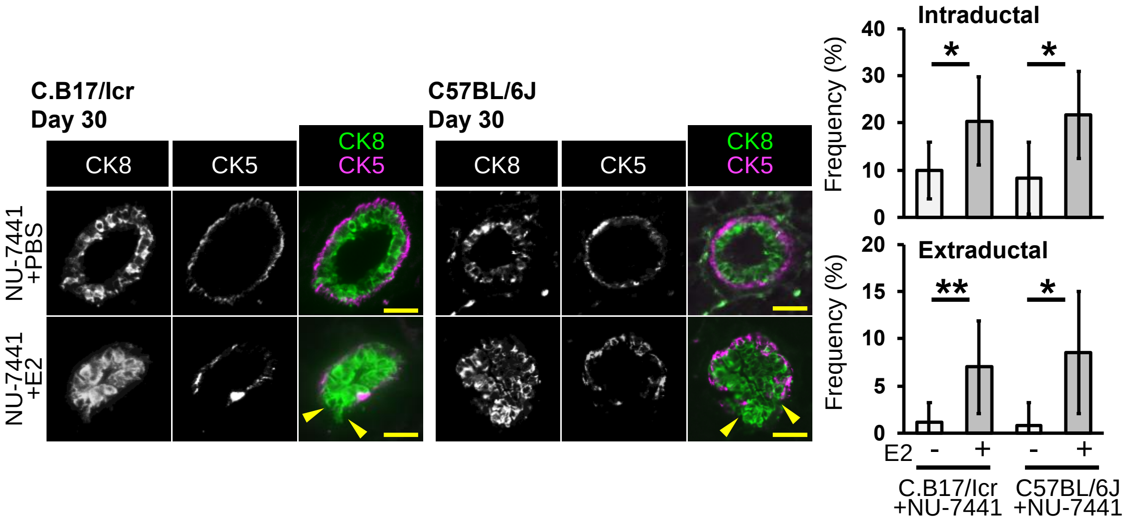

Figure S3 Dysplasia is induced by the combination of E2 administration and DNA-PK pharmacological inhibition. Related to Figure 1

Fluorescent images of CK8 and CK5 staining are shown. A DNA-PK inhibitor, NU-7441, was administered to wild-type stains, C.B-17/Icr and C57BL/6J, in combination with or without E2. Mammary ducts with intraductal and extraductal expansion were quantified (*n*=8 mice, U Mann-Whitney test in each strain). Arrowheads indicate mammary epithelial cells in extraductal region. Scale bars indicate 30 μm. *: P<0.05, **: P<0.01. Error bars represent standard deviation. In the graphs, crosses with different colors indicated the values of different animals.

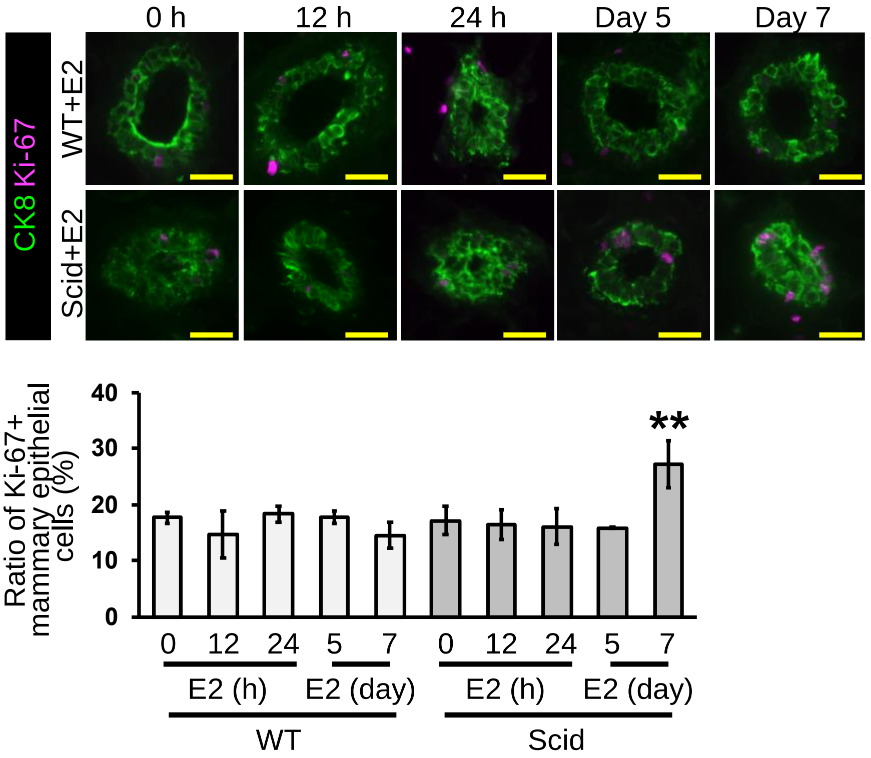

Figure S4 Mammary epithelial cell proliferation is observed at day 7 in the dysplasia model system. Related to Figure 3

Ki-67 was stained at 0 h, 12 h 24 h, 5 days and 7 days. Ratios of Ki-67-positive mammary epithelial cells were analyzed (*n*=3 mice, one-way ANOVA followed by Tukey's test). Scale bars indicate 30 µm. **: *P*<0.01. Error bars represent standard deviation. In the graph, crosses with different colors indicated the values of different animals.

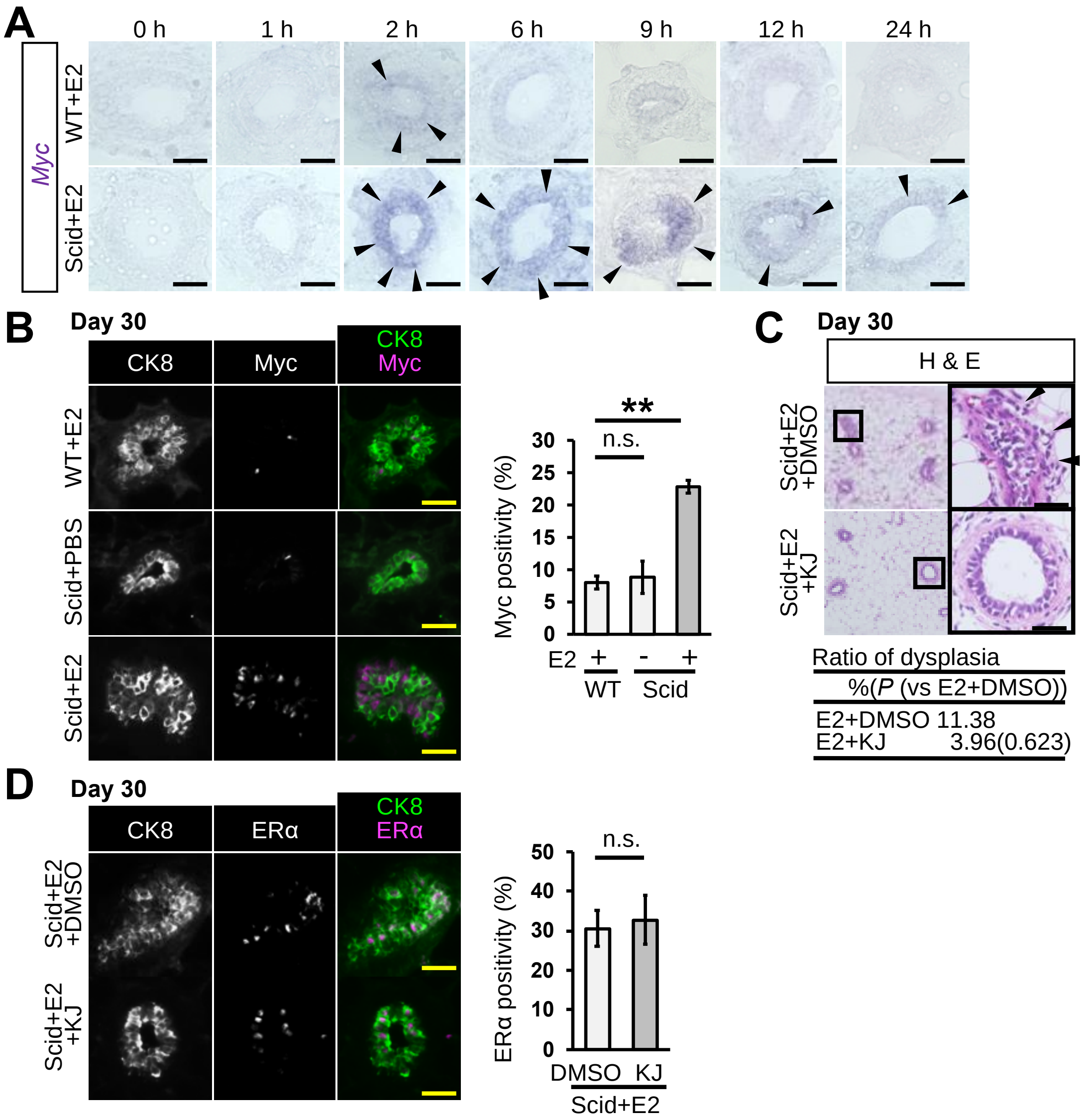

Figure S5 Estrogen administration induces Myc expression *in vivo* in mammary epithelial cells. Related to Figure 4

A, *Myc* mRNA expression was detected by *in situ* hybridization. Arrowheads indicate *Myc*-expressing domains. B, Myc expression was detected at day 30. Ratios of Myc-positive mammary epithelial cells were analyzed (n=3 mice, one-way ANOVA followed by Tukey's test). C, Typical images of H&E staining are shown. Arrowheads indicate mammary epithelial cells in extraductal region. The table shows ratios of dysplasia (n=6 mice (one image from each mouse, total 6 images), E2+DMSO 11.38% and E2+KJ 3.96% (P=0.623 (vs E2+DMSO)), U Mann-Whitney test). D, ERα expression was detected at day 30. Ratios of ERα-positive mammary epithelial cells are analyzed (n=3 mice, student's t-test). KJ: KJ-Pyr-9. Scale bars indicate 30 μm (A-D). n.s.: not significant, **: P<0.01. Error bars represent standard deviation. In the graphs, crosses with different colors indicated the values of different animals.

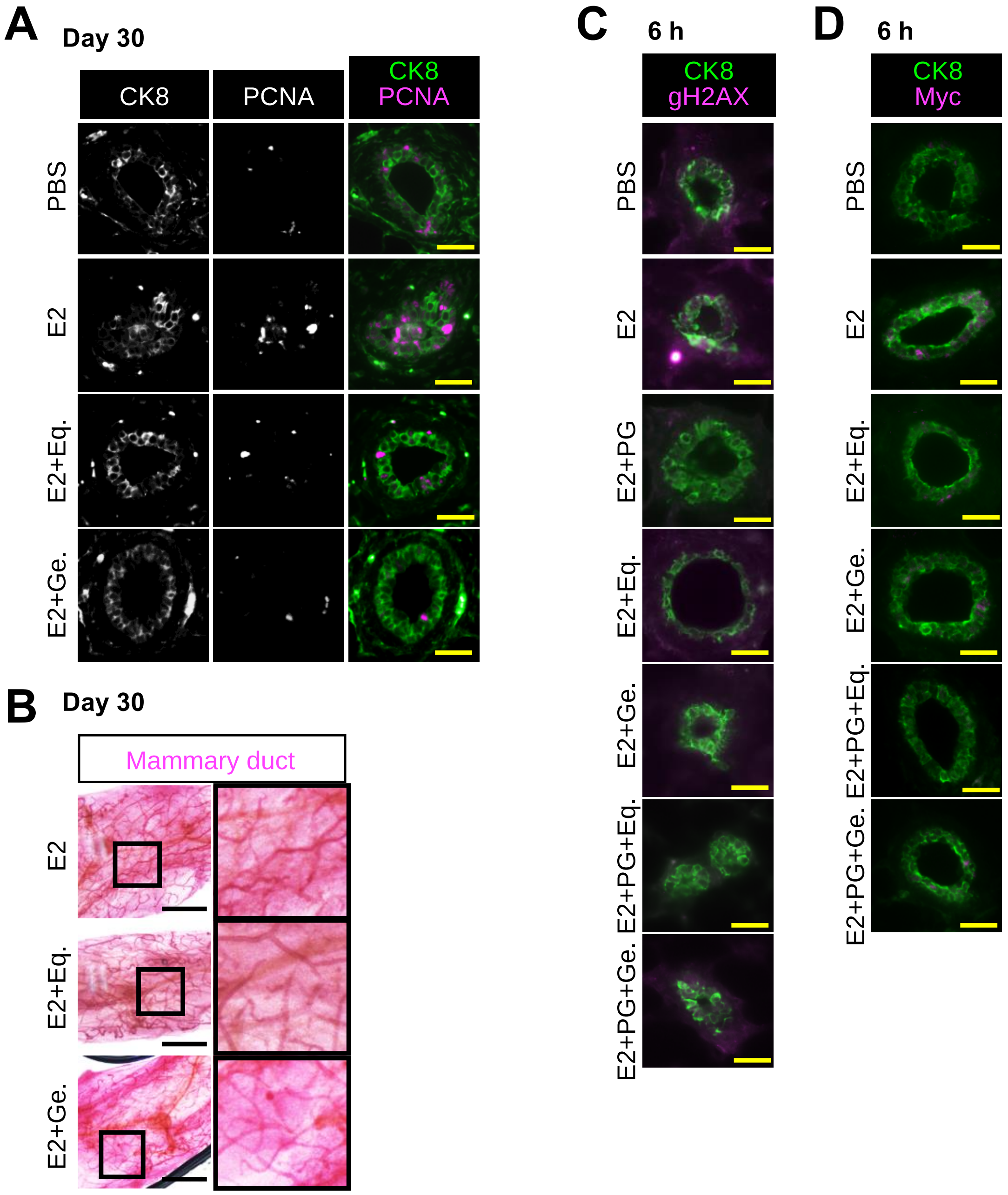

Figure S6 Isoflavones inhibit estrogen-induced cell proliferation in the mammary gland. Related to Figure 5

A, Typical images of PCNA staining are shown. Quantification is shown in Fig. 5C. B, Typical images of Carmine Alum-staining are shown. Numbers of branching are graphed in Fig. 5D. C, Gamma-H2AX was stained. Ratios of gH2AX-positive mammary epithelial cells are graphed in Fig. 5G. D, Myc immunostaining was performed. Ratios of Myc-positive mammary epithelial cells are shown in Fig. 5H. Eq.: (S)-equol, Ge.: genistein. Scale bars indicate 30 μm (A, C, D) and 2 mm (B).

Table S1 Frequency of dysplasia formation, related to Figure 1, S3

Mouse	No of mice	Total no of mammary ducts analyzed	intraductal expansion (mean(%) ±S.D.)	extraductal expansion (mean(%) ±S.D.)
C.B-17/Icr WT+E2 30d	10	232	9.68±7.53	0.34±1.09
C.B-17/Icr Scid+PBS 30d	10	235	9.48±6.89	1.14±1.85
C.B-17/Icr Scid+E2 30d	10	283	20.65±9.99	5.92±3.80
C.B-17/Icr WT+NU+PBS 30d	8	204	9.91±6.02	1.12±2.10
C.B-17/Icr WT+NU+E2 30d	8	201	20.35±9.37	6.99±4.87
C57BL/6J+NU+PBS 30d	8	128	8.40±7.62	0.83±2.35
C57BL/6J+NU+E2 30d	8	116	21.76±9.29	8.55±6.48

NU: NU-7441 (DNA-PK inhibitor)
